# Inflammatory pain in mice induces light cycle-dependent effects on sleep architecture

**DOI:** 10.1038/s41386-025-02152-w

**Published:** 2025-06-22

**Authors:** Dominika J. Burek, Khairunisa Mohamad Ibrahim, Andrew G. Hall, Ashish Sharma, Jessica A. Cucinello-Ragland, Erik S. Musiek, Jose A. Morón, William A. Carlezon

**Affiliations:** 1 Basic Neuroscience Division, McLean Hospital, Belmont, MA 02478 USA; 2 Department of Psychiatry, Harvard Medical School, Boston, MA 02115 USA; 3 Department of Anesthesiology, Washington University Pain Center, St. Louis, MO 63110 USA; 4 Washington University in St. Louis, School of Medicine, St. Louis, MO 63110 USA; 5 Department of Neurology, Washington University School of Medicine, St. Louis, Missouri 63110 USA; 6 Center on Biological Rhythms and Sleep (COBRAS), Washington University School of Medicine, St. Louis, Missouri 63110 USA; 7 Department of Neuroscience, Washington University in St. Louis, St. Louis, MO 63110 USA; 8 Department of Psychiatry, Washington University in St. Louis, St. Louis, MO 63110 USA

**Keywords:** Sleep, Neuroimmunology, Chronic pain

## Abstract

Pain syndromes include physical, sensory, emotional, and cognitive symptoms such as disability, negative affect, feelings of stress, and fatigue. Experimental induction of long-term inflammatory pain in rodents by hindpaw injection of complete Freund’s adjuvant (CFA) produces anhedonia and dysregulated naturalistic behaviors, similar to the effects of unregulated stress. We examined whether these similarities extend to changes in sleep and rhythms, such as those induced by chronic social defeat stress, using actigraphy and wireless EEG in mice. Comparisons were made between groups that received injections at the onset of the light or dark phase. We found that CFA-induced inflammatory pain alters sleep architecture in both sexes; most notably, it increased sleep duration in the dark phase—when mice are normally more likely to be awake—while also increasing sleep bout length and reducing wake bout length. In contrast, during the light phase, it decreased sleep bout length, indicating fragmentation. Similarly, CFA-induced increases in REM and SWS duration and bouts were largest during the dark phase. Dark-phase effects were remarkably consistent regardless of whether the mice had been injected at darkness onset or 12 h earlier, whereas light-phase effects were more dependent on time since injection. Injections also produced non-specific alterations in circadian rhythmicity. Our findings indicate that inflammatory pain prominently increases sleep during normally active phases as well as transitions between sleep and wakefulness throughout the day. These effects align with clinical observations and establish a basis for mechanistic studies and use of these procedures to better predict outcomes in humans.

## Introduction

An estimated 20 to 40% of Americans live with pain and report daily suffering [1, 2]. Pain can be the result of a nerve injury or inflammation, a symptom of other disorders, or idiopathic [3]. Regardless of cause, pain has profound effects on daily life: patients often report it interferes with independence, mobility, work, relationships, and sleep quality [4, 5]. Fragmented sleep and debilitating fatigue are especially burdensome for those with painful autoimmune disorders [6].

Pain can be conceptualized as a persistent stressor. The emotional toll of feelings like catastrophizing, helplessness, and despair perpetuates cycles of stress and pain [7, 8]. Approximately 30% pain patients meet diagnostic criteria for psychiatric disorders commonly associated with stress, such as Major Depressive Disorder (MDD) [9, 10]. Sleep dysregulation is a prominent feature of depression, and includes reduced latency to rapid-eye-movement (REM) sleep and increased REM duration, which are well-characterized biomarkers of MDD [11, 12]. We previously reported that the mouse model chronic social defeat stress (CSDS) promotes both REM and slow-wave sleep (SWS) [11]. The same CSDS regimen produces anhedonia—a key diagnostic feature of MDD—that develops over a virtually identical time course [13]. Linking these signs mechanistically, we showed that neural manipulations that regulate motivation also regulate sleep patterns [14]. Even acute stressors such as a single instance of social conflict or restraint have also been shown to affect sleep in humans and rodents [15–17].

This overlap among pain, stress, and sleep dysregulation may be attributable to how these experiences engage common neural circuits, including the hypothalamic-pituitary-adrenal (HPA) axis and the mesolimbic pathway [14, 18–20]. The immune system may also contribute, particularly in the case of autoimmune or inflammatory pain [21]. Pro-inflammatory signaling molecules such as cytokines IL-1β, IL-6, and TNF-α are circadian-regulated and bidirectionally modulate sleep in both illness and healthy conditions [6, 21]. Cytokines are also stimulated by CSDS and inflammatory pain, raising the possibility of shared mechanisms [22–25].

The present studies were designed to characterize the effects of inflammatory pain on sleep and to evaluate possible overlap with the effects of stress. We hypothesized that inflammatory pain would have similar effects on sleep as stress [11, 14]. To model persistent inflammatory pain, we used Complete Freund’s Adjuvant (CFA)-induced inflammation in mice. CFA comprises mycobacterium antigen, which evokes inflammation at the site of injection and throughout the central nervous system [24, 26, 27]. When injected into a hindpaw, CFA causes swelling, erythema, and thermal and mechanical hypersensitivity for up to six weeks [28]. Although numerous reports describe the behavioral phenotype of CFA-induced inflammatory pain, our preclinical meta-analysis indicated that its effects in most classical assays of anxiety- and depressive-like behaviors are small and heterogeneous [29]. Interestingly, the most prominent effects are observed in more naturalistic behaviors such as wheel running, nesting, burrowing, and in translationally-relevant signs such as anhedonia in reward-seeking behavior and motivational tasks [18, 30–33]. Since classical phenotyping assays have not yet identified strong biomarkers that enabled development of novel therapeutics, more objective and continuous measures may provide a better understanding of how pain acts as a stressor [34]. To understand the nuanced ways in which CFA-induced inflammatory pain affects sleep, we leveraged locomotor activity tracking, piezoelectric sensors, and EEG recordings. Our studies reveal that CFA-induced inflammatory pain strongly promotes sleep, causing suppression of wakefulness, reductions in wake bout length, and corresponding increases in duration and bouts of REM and SWS. Furthermore, we discovered that CFA-induced inflammatory pain promotes sleep most during periods when mice are normally active.

## Methods

### Animals

Male and female C57BL/6 J mice, 7 weeks old, were obtained from Jackson Laboratories (Bar Harbor ME, USA) and acclimated to the vivarium for one week. Mice were housed in temperature- (21 ± 2 °C) and humidity- (50 ± 20%) controlled rooms with food, water, and nesting material available *ad libitum*. Cages (except for the actigraphy experiment) were changed weekly. Procedures were approved by the Washington University in St. Louis Institutional Animal Care and Use Committee and/or the McLean Hospital Institutional Animal Care and Use Committee and performed in accordance with the National Institutes of Health’s (NIH) Guide for the Care and Use of Animals.

### CFA Injection

For actigraphy-based quantification of circadian locomotor activity and of sleep and wake states, mice were gently scruffed (held by the skin at the back of the neck) for injections. For EEG- and EMG-based quantification experiments, mice implanted with wireless transmitters could not be scruffed without damaging biopotential leads. In these experiments, mice were briefly exposed to a droplet of isoflurane in an empty cage until immobile, which enabled handling for injections. Mice began moving within 30 seconds after injection. Mice received 50 μL CFA (Sigma Aldrich, St. Louis MO, USA) at a concentration of 1.0 mg/mL or sterile 0.9% saline into the plantar surface of the hindpaw. Previous reports indicate that this dose and volume of CFA induces thermal hyperalgesia and mechanical hypersensitivity that persists for weeks [28]. For consistency with previous reports, we injected saline into the left and CFA into the right hindpaws [18, 28, 35].

### Electronic von Frey test for mechanical allodynia

To confirm previous reports of the long-lasting effects of CFA injections on hypersensitivity and inflammation, we conducted the von Frey test and caliper measurements in a separate cohort of mice not implanted with sleep transmitters [28]. Mechanical sensitivity was assessed before and at various time points after saline or CFA injections. Mice were placed in elevated enclosures (3.81 cm × 11.43 cm × 11.43 cm) with clear Plexiglas walls and a metal rod floor. Following a 15-min acclimatization period, an electronic von Frey filament (MouseMet, TopCat Metrology Ltd, UK) was applied to the mid-plantar surface of each hindpaw. The force threshold required to elicit a withdrawal response was recorded; three measurements were taken at 5-min intervals and averaged at each time point including baseline.

### Caliper measurement for hindpaw swelling

Hindpaw swelling in mm was measured with a digital caliper (Uline, Pleasant Prairie WI, USA) and photographed immediately after measurement.

### Actigraphy-based quantification of circadian locomotor activity in Light-Dark and Dark-Dark cycles

Mice were individually housed in Ancare N10 mouse cages (Ancare Corp., Bellmore, NY) equipped with passive infrared sensor wireless nodes (Actimetrics, Wilmette, IL, USA). Daily locomotor activity was recorded at 1-min intervals using the ClockLab Wireless data collection program v4.129 (Actimetrics,) under a 12 h light/12 h dark (LD) cycle (lights on from 0600, zeitgeber time [ZT] 0, to 1800 h; 300 lux) for 7 days baseline, followed by 7 days post-saline injection and then 7 days post-CFA injection. In Dark-Dark circadian locomotor activity studies, activity was recorded daily under 12 h light/12 h dark (LD) cycle (lights on from 0600 [ZT 0] to 1800 h [ZT 12]; 300 lux) for the first 6 days, followed by constant dark (DD) conditions for the next 15 days (1 additional day baseline, 7 days post-saline and 7 days post-CFA). Baselines and all measures after saline or CFA injection were averaged over 7 days. Period length (tau) was determined through χ2 periodogram analysis, rhythm strength was measured using relative Fast Fourier Transform (FFT) analysis, and other non-parametric features of circadian locomotor activity were assessed with ClockLab Analysis v6.1.02 (Actimetrics). Routine animal husbandry during the dark phase was conducted in red light ( < 10 lux). All hindpaw injections were done 3 h after “lights-on” (0900 h, ZT 3) for the LD cycle or at 0900 h (ZT 3) for the DD cycle.

### Actigraphy-based quantification of sleep and wake states

To assess sleep-like behavior with actigraphy, we used an automated piezoelectric sleep monitoring system (Adapt-A-Base, Model #MB-ACN10, Signal Solutions, Lexington, KY), which uses a 2-s pressure signal segment to determine whether the mice are awake or asleep [36, 37]. When mice are asleep, the main pressure changes are due to breathing, producing an accurate respiratory trace. In contrast, the wakefulness state exhibited irregular, transient, high-amplitude pressure changes linked to body movements and weight shifting. Even during “quiet rest,” slight head or other movements were enough to differentiate rest from sleep with accuracy comparable to EEG/EMG [37]. Mice were individually housed in cages (Ancare) and placed on the Adapt-A-Base systems in sound- and light-proof circadian cabinets (Actimetrics). They were kept under a 12 h light/12 h dark cycle. Each cage contained 160 g of corncob bedding, 220 g of food pellets, and 380 mL of water, ensuring consistent cage weight except for minor variations in mouse body weight. Sleep/wake states were recorded continuously for 15 days using the PiezoSleep v2.08 program (Signal Solutions) without any disturbance except for saline or CFA hindpaw injections, which were done 3 h after “lights-on” (0900 h, ZT 3). Baselines were averaged over 2 days. Sleep data were analyzed using SleepStats v2.18 (Signal Solutions). Sleep or wake bout lengths were scored in 30-second epochs.

### EEG- and EMG-based quantification of sleep telemetry: implant surgeries

To assess sleep behavior in mice with physiological measurements, we implanted mice with HD-X02 wireless telemetry devices (DSI; St. Paul, MN) to enable continuous recording of electroencephalograms (EEG), electromyogram (EMG), locomotor activity, and subcutaneous temperature. Surgical procedures were performed as described previously [38]. Briefly, mice were anesthetized with 2% isoflurane and immobilized on a stereotaxic instrument. A small incision was made from the medial skull to posterior neck, through which a subcutaneous pocket on the back was opened using lubricated forceps. Transmitters were inserted lateral to the spine, midway between the fore and hind limbs. Two incisions were made in the trapezius muscle using a 21 G needle, through which two insulated biopotential leads (EMG electrodes) were threaded and secured using 6.0 non-dissolvable silk sutures. EEG biopotential leads were attached to two stainless steel screws, inserted into the skull, and lowered until they contacted dura (relative to bregma: frontal AP + 1.0 mm, ML + 1.0 mm; parietal AP − 3.0 mm, ML − 3.0 mm). Screws and EEG leads were secured with dental cement and the incision was closed with 6.0 non-dissolvable sutures. Triple antibiotic ointment and 2% lidocaine were applied topically to the incision site. Ketoprofen (5 mg/kg, subcutaneous) was administered immediately after surgery, and once daily for 7 days of recovery.

### EEG- and EMG-based quantification of sleep telemetry: data acquisition & physiological recordings

Mice were single housed in N10 mouse cages under a standard 12 h light/12 h dark cycle. Cages sat on RPC-1 PhysioTel receiver platforms (DSI), which detect signals emitted by the HD-X02 transmitter. Receivers connected to a data exchange matrix that continuously uploaded recording data (sampling rate 500 Hz) to Ponemah Software (DSI). Data were analyzed with Neuroscore Software V3.4 (DSI). Raw EEG signal was quantified into relative spectral power in 10-second epochs with FFT and a Hamming signal window. Frequency bands were defined as delta (0.5–4 Hz), theta (4–8 Hz), alpha (8–12 Hz), beta (16–24 Hz), and low gamma (30–50 Hz). Vigilance states of active wakefulness (AW), REM sleep, and SWS were assigned using an automated Neuroscore algorithm, calibrated to each baseline recording. For any 10-s epoch, AW required more than 20% of EEG power to be above the maximum amplitude recorded during either stage of sleep; REM required largest peak in theta power, theta power to be at least 1.1x that of delta power, and minimum EMG amplitude; SWS required largest peak in delta power, delta power to be on average at a ratio of 0.3 to the power of theta, alpha, beta, and gamma combined, and minimum EMG amplitude (see Fig. S1 for a representative image). Total duration and uninterrupted bouts of each stage were calculated separately for 12 h of lights-on and 12 h of lights-off per 24-h period. Body temperature and activity counts were automatically provided by Neuroscore. Activity was normalized to wake duration by dividing activity counts by wake duration, to account for differences in wake duration across time. Baseline was averaged from 6 days of recording, then used to normalize measures within-subjects for recordings after isoflurane exposure, saline injection, and CFA injection.

### Statistical analyses

Statistical analyses were performed using Prism 9 (GraphPad, Boston, MA) with significance set to *p* < 0.05. We first performed analyses on pooled (male and female) mice, as described [39]. N’s=6 per sex were used for the electronic von Frey test and caliper measurements; N’s=6 per sex for actigraphy-based quantification of circadian locomotor activity; N’s=8 per sex for actigraphy-based quantification of sleep and wake states, and N’s 4-6 per sex were used for EEG- and EMG-based quantification of sleep and wake states. We also ran secondary analyses comparing sexes and report statistically significant differences (when applicable). Measures for pooled sexes were assessed for normality with Kolmogorov-Smirnov tests, compared with *t*-tests (normal distributions) or nonparametric Wilcoxon matched-pairs signed rank tests (non-normal distributions), repeated measures one-way ANOVAs and *post hoc* Tukey’s multiple comparisons (normal distributions) or nonparametric Friedman tests followed by *post hoc* Dunn’s tests (non-normal distributions), and compared to a theoretical mean of 100 in within-group *t*-tests or nonparametric Wilcoxon signed rank tests. Measures comparing males and females were compared with repeated measures two-way ANOVAs, Tukey’s and Sidak’s multiple comparisons, and means for each sex individually compared to a theoretical mean of 100 in within-group *t*-tests. For clarity and brevity, nonsignificant ANOVA and post-hoc results are not reported in the main text. ROUT testing identified two outliers: one excluded from Fig. 3B; and one excluded from Fig. 3H. Exclusion of these data does not change statistical outcomes. In longitudinal analyses of CFA’s effect on sleep measures in the dark phase (Figs. S2, S4) cage changes on the last day of each week drastically increased wake duration; these days were excluded in analyses. Transmitter batteries failed in two female mice on CFA days 10 and 11, necessitating use of a mixed-effects model ANOVA. Finally, radar plots (Figs. 4F, K, 5F, K) were generated with the FMSB package in RStudio (Version 2022.07.2 + 576). To qualitatively depict group differences, the middle of each variable’s axis range was set to the saline control group average for that individual endpoint. Detailed statistical analysis and sample size are reported in the supplementary file.

## Results

Studies were conducted in both males and females to identify sex differences. Occasional sex differences were observed, frequently without systematic patterns. All such instances are described.

### CFA induces persistent swelling and mechanical hypersensitivity

**Fig. 1:**
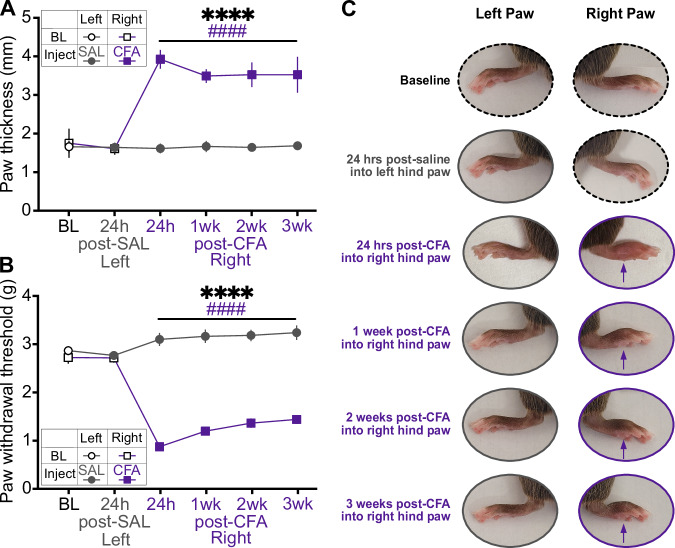
Indicators of the inflammatory effect of CFA compared to baseline, 24 h after saline injection, and 24 h, 1 week, 2 weeks, and 3 weeks after CFA injection. **A** Hindpaw swelling, measured as increased thickness in mm with a digital caliper (Time x Paw interaction: F_(5,55)_ = 122.8, p < 0.0001; Sidak’s *post hoc* tests assessing between-groups differences in paw thickness at 24 h post-CFA: *p* < 0.0001). **B** Mechanical hypersensitivity, measured by reduced force required in grams for paw withdrawal (Time x Paw interaction: F_(5,55)_ = 49.75, *p* < 0.0001; between-groups comparisons, *p* < 0.0001). **C** Representative images of each paw in a single mouse. Purple arrows indicate CFA-induced inflammation. *indicates a significant main effect of time (****p < 0.0001). ^#^indicates a significant difference from baseline value (^####^*p* < 0.0001).

We first confirmed that within-subjects comparisons between saline injection into the left hindpaw and CFA injection into the right hindpaw resulted in acute and persistent CFA-specific inflammation (Fig. 1). Hindpaw thickness and mechanical hypersensitivity (allodynia) were measured at baseline, 24 h after saline injection into the left hindpaw, 24 h after CFA injection into the right hindpaw, and weekly for 3 weeks after. While there were no differences between hindpaws at baseline or after saline injection, CFA induced dramatic swelling that persisted for three weeks (Fig. 1A, C). Similarly, CFA reduced the force required for hindpaw withdrawal, an effect that also lasted for three weeks (Fig. 1B, C). These results confirm our previous findings describing the rapid onset and long persistence of CFA-induced inflammatory pain, and indicate our actigraphy and physiological recordings covered periods of inflammation and associated mechanical sensivity [28].

### CFA-induced inflammatory pain does not alter circadian rhythms

**Fig. 2:**
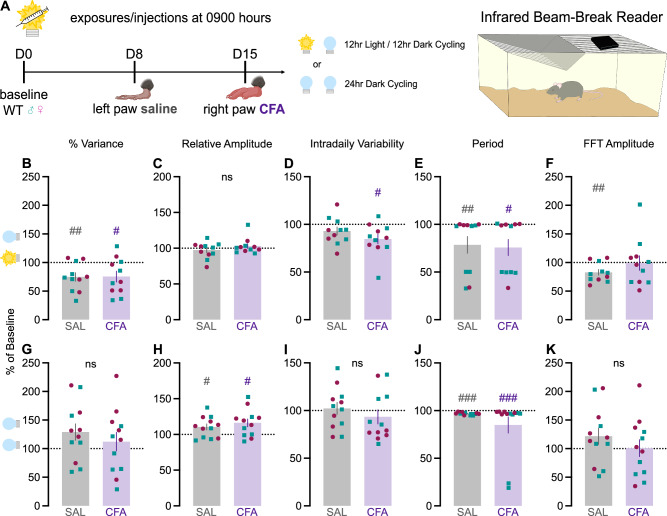
Actigraphy-based quantification of circadian locomotor activity in light-dark and dark-dark cycles. **A** Mice were individually housed in cages equipped with passive infrared sensor wireless nodes. Daily locomotor activity was recorded for 7 days of baseline, 7 days after saline injection, and 7 days after CFA injection. As percent of average baseline (dotted line at 100%), **B** percent variance (between-groups comparisons: t_10_ = 0.08684, *p* = 0.9325; within-group comparisons to 100% baseline SAL: t_10_ = 3.420, *p* = 0.0066; within-group CFA: t_10_ = 2.595, *p* = 0.0267), **C** relative amplitude (between-groups Wilcoxon for non-normal distributions: *p* = 0.1016), **D** intradaily variability (between-groups: t_10_ = 2.094, *p* = 0.0627; within-group CFA: t_10_ = 3.009, *p* = 0.0131), **E** period (between-groups Wilcoxon: *p* = 0.9658; within-group SAL: *p* = 0.0010; within-group CFA: *p* = 0.0010), and **F** FFT amplitude (between-groups: t_10_ = 1.231, *p* = 0.2466; within-group SAL: t_10_ = 3.436, *p* = 0.0064; within-group CFA: t_10_ = 0.07744, *p* = 0.9398) under 12 h light and 12 h dark cycling conditions. As percent of average baseline, **G** percent variance (between-groups: t_11_ = 1.404, *p* = 0.1878), **H** relative amplitude (between-groups: t_11_ = 2.035, *p* = 0.0667; within-group SAL: t_11_ = 2.510, p = 0.0290; within-group CFA: t_11_ = 2.954, *p* = 0.0131), **I** intradaily variability (between-groups: t_11_ = 1.995, *p* = 0.0715), **J** period (between-groups Wilcoxon: *p* = 0.7695, within-group SAL: *p* = 0.0005; within-group CFA: *p* = 0.0005), and **K** FFT amplitude (between-groups: t_11_ = 1.844, *p* = 0.0923) under 24 h constant dark conditions. Teal squares indicate males and magenta circles indicate females. Ns indicates not significant. ^#^indicates significant difference from theoretical baseline of 100% (^#^*p* < 0.05, ^##^*p* < 0.01, ^###^*p* < 0.001).

We next assessed whether CFA injection affects circadian rhythms via locomotor activity, which was recorded using passive infrared sensor wireless nodes for 7 days of baseline, 7 days after saline injection, and 7 days after CFA injection (Fig. 2A). Relative to baseline, both saline and CFA reduced percent variance, an indirect measure of daily phase stability, indicating less repetitive and more unstable patterns on a daily basis (Fig. 2B). Neither saline nor CFA altered relative amplitude, which is the ratio of the most active 10 h to the least active 5 h (Fig. 2C), with greater relative amplitude indicating a robust 24-h rest-activity (circadian) rhythm. CFA reduced intra-daily variability, a measure of 24-h rhythm fragmentation, from baseline, though this effect did not differ significantly from saline (Fig. 2D). This measure reflects how often activity transitions between high and low levels, and our findings indicate CFA resulted in longer periods of rest and activity, potentially due to brief bouts of sleep during the active phase or deconsolidated sleep. For some mice, both saline and CFA significantly reduced the circadian period—time difference between two peaks or troughs of locomotor activity—from an approximate 24-h baseline (Fig. 2E), suggesting a non-specific effect of the injections themselves. Surprisingly, saline, but not CFA, reduced the difference between the highest point of locomotor activity and the mean activity of the rhythm as calculated by FFT amplitude, indicating less variation in locomotor activity across the circadian period (Fig. 2F). Collectively, these findings suggest that CFA did not have robust or consistent effects on circadian rhythms.

We then repeated the experiment using a constant darkness protocol to determine if effects of CFA on circadian rhythms might be masked by entrainment to the 12h-light/12h-dark cycle. In the dark-dark protocol, mice were tracked for 6 days of baseline under regular light-dark conditions before switching to constant dark conditions 24 h prior to saline injection. Neither saline nor CFA produced significant effects on percent variance (Fig. 2G), intra-daily variability (Fig. 2I), or FFT amplitude (Fig. 2K). Both saline and CFA increased relative amplitude (Fig. 2H) and significantly decreased the period (Fig. 2J). When considered altogether, these findings indicate that CFA did not dramatically alter circadian rhythms during the 2 weeks of inflammatory pain, regardless of the light cycle.

### CFA-induced inflammatory pain increases sleep duration and decreases wake bout length

**Fig. 3:**
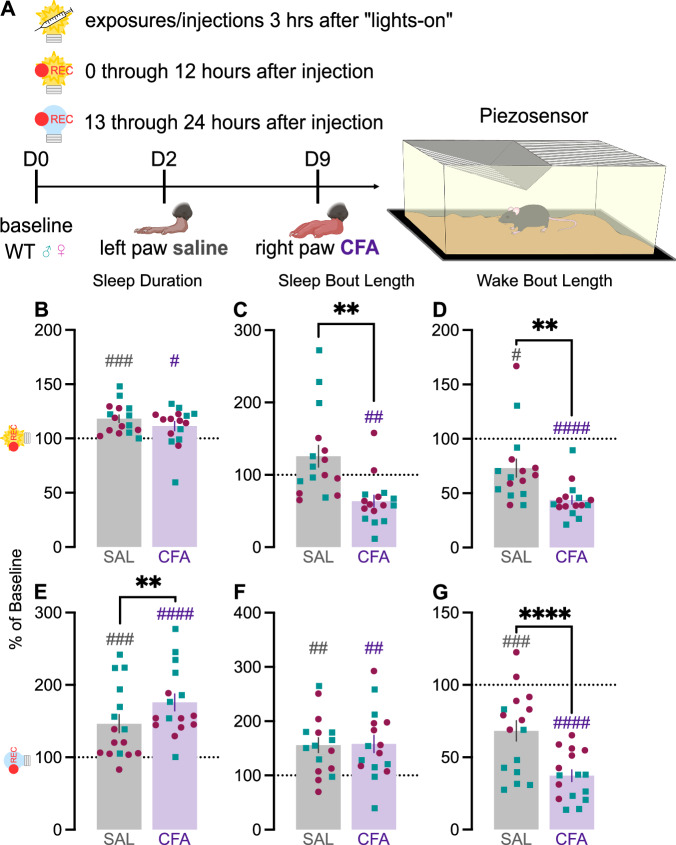
Actigraphy-based quantification of sleep and wake states. **A** Mice were individually housed in cages on automated piezoelectric sleep monitoring systems. Sleep and wake behavior were recorded for 2 days of baseline, 7 days after saline injection, and 7 days after CFA injection. Data presented here is for the first 24 h after each injection. As percent of average baseline (dotted line at 100%), **B** sleep duration (between-groups: t_15_ = 1.629, *p* = 0.1242; within-group SAL: t_15_ = 5.192, *p* < 0.0001; within-group CFA: t_15_ = 2.592, *p* = 0.0204), **C** sleep bout length (between-groups Wilcoxon: *p* = 0.0013; within-group SAL: *p* = 0.3225; within-group CFA: *p* = 0.0027), and **D** wake bout length (between-groups Wilcoxon: *p* = 0.0042; within-group SAL: *p* = 0.0155; within-group CFA: *p* < 0.0001) during lights-on. As percent of average baseline **E** sleep duration (between-groups Wilcoxon: *p* = 0.0027; within-group SAL: *p* < 0.0004; within-group CFA: *p* < 0.0001), **F** sleep bout length (between-groups: t_15_ = 0.1013, *p* = 0.9207; within-group SAL: t_15_ = 4.1071, *p* = 0.0010; within-group CFA: t_15_ = 3.652, *p* = 0.0024), and **G** wake bout length (between-groups: t_15_ = 5.745, *p* < 0.0001; within-group SAL: t_15_ = 4.485, *p* = 0.004; within-group CFA: t_15_ = 15.34, *p* < 0.0001) during lights-off. Teal squares indicate males and magenta circles indicate females. Ns indicates not significant. ^#^indicates significant difference from theoretical baseline of 100% (^#^*p* < 0.05, ^##^*p* < 0.01, ^###^*p* < 0.001, ^####^*p* < 0.0001). *indicate significant group differences (*p < 0.05, **p < 0.01, ***p < 0.001).

Although CFA did not reliably impact circadian rhythm, some of the metrics suggested it may alter sleep quality. To assess this possibility, we examined whether CFA affects sleep quality using an automated piezoelectric sleep monitoring system to detect breathing and gross body movements, which can be used to estimate sleep and wake patterns. Mouse activity was recorded for 2 days of baseline, 7 days after saline injection, and 14 days after CFA injection in standard light-dark conditions (Fig. 3A). In the first 24 h after injection, saline and CFA both increased sleep duration in the light phase (hours 1–12 after the injection) relative to baseline (Fig. 3B). While saline caused nominal (non-significant) increases in sleep bout length in the light phase, CFA significant reduced it (Fig. 3C). Wake bout length in the light phase was also reduced by both saline and CFA, with the effect being more pronounced following CFA (Fig. 3D). In the dark phase (hours 13–24 after the injection), both saline and CFA again significantly increased sleep duration, the increase was significantly greater with CFA (Fig. 3E). In contrast to effects seen during the light phase, CFA increased sleep bout length in the dark phase (Fig. 3F). As in the light phase, wake bout length in the dark phase was also reduced by both saline and CFA, and more robustly by CFA (Fig. 3G).

While sleep duration in the light phase remained consistently elevated for both males and females across two weeks (Fig. S2A), sleep bout length increased over this time (Fig. S2B). Light-phase wake bout length increased over two weeks but remained below baseline (Fig. S2C). In the dark phase, sleep duration was elevated most for females in the first week after CFA injection, returning close to baseline during the second week (Fig. S2D). Dark-phase sleep duration was elevated more for females than males in the first week after injection, but both sexes recovered close to baseline in the second week (Fig. S2E). Wake bout length rose steadily, with males showing recovery sooner, but did not go back to baseline for either sex even in the second week (Fig. S2F). When considered altogether, these findings indicate that CFA increased sleep duration and reduced wake bout length. While saline alone also affected some parameters of circadian rhythmicity and sleep-wake behavior, this is most likely a transient effect of restraint, injection, and fluid being injected into and briefly distending the hindpaw. Considering that there was no lasting effect of saline injection on hindpaw size or allodynia during the recording period, the significant differences between the saline injection and the CFA injection a week later indicate a specific effect of inflammatory pain.

### CFA-induced inflammatory pain promotes increased but fragmented REM and SWS

**Fig. 4:**
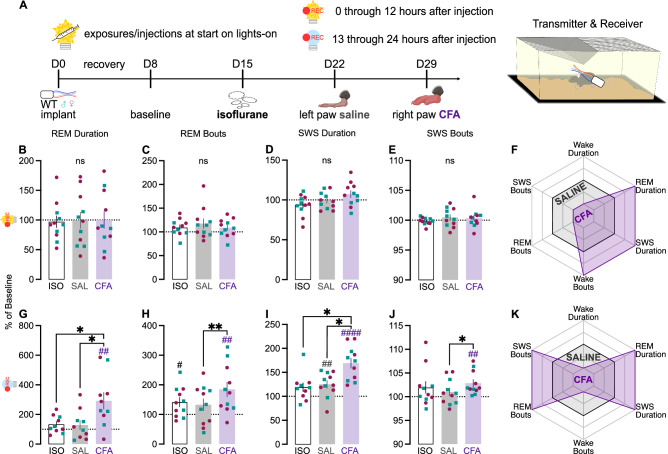
EEG- and EMG-based quantification of sleep telemetry after CFA injection during lights-on. **A** Mice were implanted with wireless telemetry transmitters. EEG and EMG were recorded for 7 days of baseline, 7 days after isoflurane exposure, 7 days after saline injection, and 21 days after CFA injection. Data presented here is for the first 24 h after each injection. Mice were injected at the beginning of the light phase. As percent of average baseline (dotted line at 100%), **B** REM duration (F_(1.986,19.86)_ = 0.02847, *p* = 0.9714), **C** REM bout number (F_(1.427,14.27)_ = 0.745, *p* = 0.4489), **D** SWS duration (F_(1.390,13.90)_ = 4.341, *p* = 0.0457; secondary analysis of sexes individually: Time x Sex interaction: F_(2,18)_ = 6.197, *p* = 0.0090; Female ISO vs. CFA: *p* = 0.0133; SAL vs. CFA: *p* = 0.0302), and **E** SWS bout number (F_(1.960,19.60)_ = 0.8844, *p* = 0.4269) during lights-on. **F** Summary radar plot indicating direction of change between saline and CFA for lights-on. As percent of average baseline, **G** REM duration (F_(1.446,13.01)_ = 10.72, *p* = 0.0032, ISO vs. CFA *p* = 0.0224, SAL vs. CFA *p* = 0.0115; within-group CFA: t_9_ = 3.462, *p* = 0.0071), **H** REM bout number (F_(1.716,17.16)_ = 6.088, *p* = 0.0126, SAL vs. CFA *p* = 0.0092; within-group ISO: t_10_ = 2.740, *p* = 0.0208; within-group CFA: t_10_ = 3.455, *p* = 0.0062; secondary analysis of sexes individually: main effect of Time: F_(1.489,13.40)_ = 6.893, *p* = 0.0130; Male SAL vs. CFA *p* = 0.0443), **I** SWS duration (F_(1.976,28.66)_ = 8.823, *p* = 0.0011, ISO vs. CFA *p* = 0.0112, SAL vs. CFA p = 0.0170; within-group SAL: t_10_ = 3.188, *p* = 0.0097; within-group CFA: t_10_ = 6.548, *p* < 0.0001), and **J** SWS bout number (Friedman test F = 6.545, *p* = 0.0435, SAL vs. CFA *p* = 0.0315; within-group Wilcoxon CFA: *p* = 0.0010) during lights-off. **K** Summary radar plot indicating direction of change between saline and CFA for lights-off. Teal squares indicate males and magenta circles indicate females. Ns indicates not significant. ^#^indicates significant difference from theoretical baseline of 100% (^#^*p* < 0.05, ^##^*p* < 0.01, ^####^*p* < 0.0001). *indicate significant group differences (*p < 0.05, **p < 0.01).

To more comprehensively characterize the effect of CFA on sleep, we investigated whether the increased sleep was attributable to REM, SWS, or both using wireless telemetry transmitters to enable untethered and continuous recording of EEG, EMG, locomotor activity, and subcutaneous temperature (Fig. 4A). EEG and EMG were recorded for 7 days of baseline, 7 days after isoflurane exposure (to control for the anesthesia required to inject transmitter-implanted mice), 7 days after saline injection, and 21 days after CFA injection. In separate cohorts of mice, injections occurred either at the onset of the light phase or the onset of the dark phase, to enable assessment of potential contributions of testing during periods when the mice are normally more or less likely to be active. Because these studies required brief ISO anesthesia to perform the hindpaw injections without risking damage to the subcutaneous EEG/EMG leads, comparisons include an ISO alone condition.

Initial analyses focused on the 24 h after CFA injection was given at the beginning of the light phase (when mice are less likely to be active). First, we confirmed that CFA reduces active wake (AW) duration during the light phase when sexes were pooled. Secondary analyses of the sexes individually revealed significant effects of CFA on AW duration in females (Fig. S3A). The AW bouts were unaffected by CFA (Fig. S3B). CFA significantly decreased locomotor activity compared to baseline (Fig. S3C).

During the dark phase of this regimen, CFA had a more pronounced effect, significantly decreasing AW duration when sexes were pooled with secondary analyses of the sexes individually revealed significant effects of CFA on AW duration specifically in males (Fig. S3D). Similarly, CFA also significantly reduced AW dark phase bouts when sexes were pooled with secondary analyses indicated AW bouts were reduced specifically in males (Fig. S3E). CFA reduced locomotor activity when sexes were pooled, but secondary analyses indicated this effect was significant in females only (Fig. S3F).

Next, we assessed if the reduction in wake duration is due to changes in REM and/or SWS. During the light phase, CFA had no effect on REM duration or bouts (Fig. 4B, C). Although CFA appeared to alter SWS duration when sexes were pooled, *post hoc* analyses indicated that none of the individual comparisons reached significance. Secondary analyses of the sexes individually revealed significant effects SWS duration in females only (Fig. 4D). There were no effects on SWS bouts (Fig. 4E). In summary, in the first 12 h after injection during the light phase, CFA increased REM duration, SWS duration, and wake bouts relative to saline (Fig. 4F).

During the dark phase, however, CFA increased both REM duration (Fig. 4G) and REM bouts (Fig. 4H). REM bouts were most increased for males (Fig. 4H). CFA also affected SWS during the dark phase. SWS duration (Fig. 4I) and bouts (Fig. 4J) were both significantly increased. Thus, when the dark phase begins 12 h after injections, CFA promoted both REM and SWS sleep while suppressing wake (Fig. 4K).

Next, we determined the time course of the dark-phase effects of CFA. Consistent with our piezosensor findings (Fig. 3), EEG and EMG recordings showed that CFA injection primarily affects sleep during the first three days post-injection (Fig. S4). Wake duration, wake bouts, and REM bouts were significantly dynamic across three weeks (Fig. S4A, S4E). When considered altogether, results from this experimental design (injections at the beginning of the light phase) indicate that CFA suppresses active wakefulness, with corresponding elevations in both REM and non-REM, or slow-wave, sleep, during the dark phase when animals are typically more active.

### CFA-induced inflammatory pain promotes sleep most consistently in the dark phase

To determine whether these changes are specific to the dark phase, or if CFA requires ~12 h to produce alterations in sleep, we repeated the experiment but instead performed the injections at the onset of the dark phase. In general, the pattern of effects during the dark phase specifically was similar regardless of whether testing began in darkness immediately after the injection or 12 h later. When the tests in darkness were conducted first, CFA caused immediate reductions in active wakefulness duration (Fig. S5A) and bouts (Fig. S5B). There were no significant changes in dark phase locomotor activity (Fig. S5C). During the light phase, CFA also significantly reduced AW duration (Fig. S5D) without significant effects on AW bouts or locomotor activity (Fig. S5E, F). When considered altogether, these findings show that CFA injection at the beginning of the dark phase reduced active wake duration 13 to 24 h later, during the light phase, and mice sleep even more during what is already their inactive phase. During the dark phase, however, when mice are typically more active, CFA significantly reduced active wake duration, bouts, and bout length regardless of when the injections occurred.

**Fig. 5:**
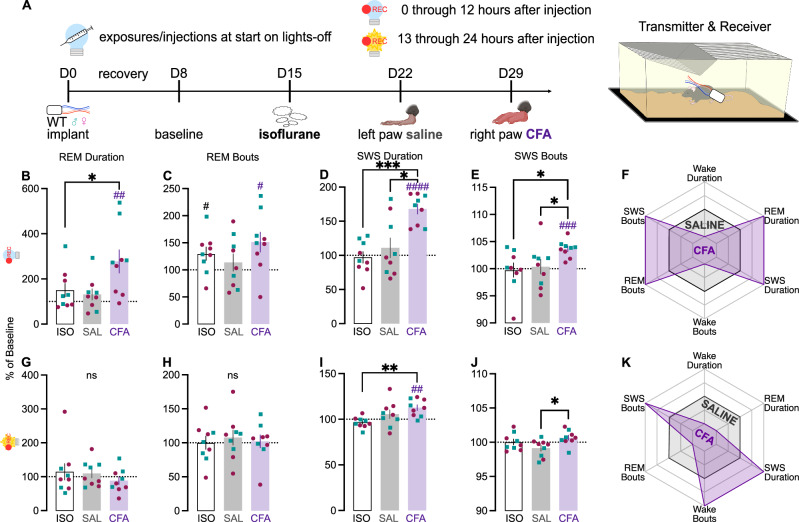
EEG- and EMG-based quantification of sleep telemetry after CFA injection during lights-off. **A** Mice were implanted with wireless telemetry transmitters. EEG and EMG were recorded for 7 days of baseline, 7 days after isoflurane exposure, 7 days after saline injection, and 21 days after CFA injection. Data presented here is for the first 24 h after each injection. Mice were injected at the beginning of the dark phase. As percent of average baseline (dotted line at 100%), **B** REM duration (F_(1.736,13.89)_ = 6.291, *p* = 0.0136, ISO vs. CFA *p* = 0.0313; within-group CFA: t_8_ = 3.466, *p* = 0.0085; secondary analysis of sexes individually: main effect of Time: F_(1.558,10.91)_ = 8.791, *p* = 0.0075, main effect of Sex: F_(1,7)_ = 6.058, *p* = 0.0434; Female ISO vs. CFA, *p* = 0.0245), **C** REM bout number (F_(1.671,13.37)_ = 2.908, *p* = 0.0961; within-group ISO t_8_ = 2.429, *p* = 0.0413; within-group CFA t_8_ = 2.794, *p* = 0.0234), **D** SWS duration (F_(1.571,12.57)_ = 15.37, *p* = 0.0007, ISO vs. CFA *p* = 0.0002, SAL vs. CFA *p* = 0.0127; within-group CFA: t_8_ = 9.234, *p* < 0.0001; secondary analysis of sexes individually: (Time F_(1.402,9.816)_ = 13.83, *p* = 0.0025; Female ISO vs. CFA *p* = 0.0020), and **E** SWS bout (F_(1.683,13.47)_ = 5.901, *p* = 0.0177, ISO vs. CFA *p* = 0.0305, SAL vs. CFA p = 0.0225; within-group CFA: t_8_ = 7.063, *p* = 0.0001) number during lights-off. **F** Summary radar plot indicating direction of change between saline and CFA for lights-off. As percent of average baseline, **G** REM duration (F_(1.240,9.919)_ = 1.053, *p* = 0.3478), **H** REM bout number (F_(1.826,14.61)_ = 0.259, *p* = 0.7555), **I** SWS duration (F_(1.948,15.58)_ = 8.956, *p* = 0.0027, ISO vs. CFA *p* = 0.0062; within-group CFA: t_8_ = 4.117, *p* = 0.0034; main effect of Time F_(1.928,13.50)_ = 8.119, *p* = 0.0051; Female ISO vs. CFA *p* = 0.0149), and **J** SWS bout number (F_(1.619, 12.96)_ = 6.095, *p* = 0.0175, SAL vs. CFA *p* = 0.0193) during lights-on. **K** Summary radar plot indicating direction of change between saline and CFA for lights-on. Teal squares indicate males and magenta circles indicate females. Ns indicates not significant. ^#^indicates significant difference from theoretical baseline of 100% (^#^*p* < 0.05, ^##^*p* < 0.01, ^###^*p* < 0.001, ^####^*p* < 0.0001). *indicate significant group differences (*p < 0.05, **p < 0.01, ***p < 0.001).

Next, we assessed whether the timing of CFA injections would differentially affect REM and SWS. REM duration during the dark phase was increased. Secondary analyses indicated CFA increased REM duration significantly for females (Fig. 5B). REM bouts were increased during the dark phase (Fig. 5C). Dark phase SWS, however, was affected more by CFA injection at the beginning of the dark phase: CFA increased SWS duration. Secondary analyses of the sexes individually revealed significant effects in females (Fig. 5D). SWS bouts in the dark phase were also increased by CFA (Fig. 5E). Thus, in the first 12 h of the dark phase immediately after injection, CFA promoted SWS and REM sleep while suppressing time spent awake (Fig. 5F). Considering that the pattern of results is similar regardless of when the dark phase occurs (compare Fig. 4K to Fig. 5F), these findings indicate the dark-phase effects seen with light-onset injections did not depend upon a 12-h pretreatment period.

During the light phase, CFA did not affect REM duration or bouts (Fig. 5G, H). However, CFA increased SWS duration in the light phase, specifically for females (Fig. 5I). Slow wave sleep bouts in the light phase were also increased with CFA (Fig. 5J). Thus, CFA promoted SWS relative to saline in the light phase, 13 through 24 h after injection (Fig. 5K).

As seen when CFA injections were given during the light phase, EEG and EMG recordings showed that CFA injection given during the dark phase primarily affected sleep during the first two to three days post-injection (Fig. S6). However, CFA administered at the onset of the dark phase resulted in far more significant effects of sex, and time x sex interactions. Wake duration recovered to baseline sooner in females, while male wake duration stay suppressed for almost three weeks (Fig. S6A). In males, REM duration remained elevated for three weeks but closer to baseline after the first week, whereas in females, REM duration dropped and stayed below baseline (Fig. S6B). In females, SWS duration recovered sooner but dropped to below baseline, while males had sustained elevations in SWS duration for three weeks (Fig. S6C). Male wake bouts were persistently lower than in females (Fig. S6D). REM bouts were elevated for the first week after CFA injection but close to baseline without sex differences thereafter (Fig. S6E). Male SWS bouts remained elevated, while female SWS dropped below baseline (Fig. S6F).

Considered altogether, these findings indicate that regardless of whether the mice were injected at the beginning of lights-on (dark phase was 12 h later) or the beginning of lights-off (dark phase was immediate), CFA produced similar effects on sleep patterns during the dark phase. Specifically, CFA-induced inflammatory pain suppresses wakefulness while causing corresponding increases in both REM and SW sleep, primarily in the dark phase when mice are typically more active. In contrast, the effects seen during the light phase differed according to the amount of time after the injections. These findings suggest that the effects on sleep may be most detectable during periods when the mice are normally more likely to be awake, and less detectable during periods when they are normally more likely to be asleep.

### CFA-inflammatory pain produces phase-dependent effects on body temperature

The wireless transmitters also provide subcutaneous body temperature recordings. We hypothesized that CFA and the increased sleep it causes would also increase temperature. Surprisingly, CFA reduced body temperature during both lights-on, particularly for females (Fig. S7A) and lights-off (Fig. S7B). In contrast, CFA injected at dark phase onset significantly increased body temperature during both the dark phase (Fig. S7C) and light phase (Fig. S7D).

Considering that the changes in body temperature differ dramatically after light- and dark-phase CFA injections, our findings suggest that thermoregulation is differentially regulated depending on when inflammatory pain is induced.

## Discussion

CFA-induced inflammatory pain profoundly alters sleep architecture in male and female mice. CFA increased sleep duration and sleep bout length during the dark phase (when mice are normally more likely to be awake and active), whereas it decreased sleep bout length in the light (when mice are more likely to be asleep and/or less active) without changing duration, indicating fragmentation. Additionally, CFA produced corresponding reductions in wake bout length, especially during the dark phase. The overall increase in sleep was due to increases in both REM and SWS, which were also most detectable in the dark phase. In fact, the effects during the dark phase were remarkably similar regardless of whether the dark phase began immediately after CFA injection or 12 h earlier. These findings suggest that the nociceptive effects of CFA become evident soon after injection and are most detectable in the dark phase because they promote sleep and/or repress activity when the mice are normally more active. While effects on sleep are also detectable during the light phase, the effects are less prominent, most likely because they are interacting with normal sleep patterns. These effects are not due to CFA-specific effects on circadian rhythms, since injection of the hindpaw (whether with CFA or saline) produced non-specific effects on circadian rhythmicity, causing reductions in variance, period, and amplitude. Considered altogether, these results (Table S1) indicate that CFA-induced inflammatory pain acutely promotes but also fragments sleep.

There are few other reports on CFA effects on sleep in rodents. In one previous report, rats injected at postnatal day 1 exhibited sleep fragmentation later in adulthood—far beyond the time scale studied here—with changes including longer sleep latency, reduced latency to REM sleep, and reduced sleep efficiency [40]. In a report describing EEG studies in adult rats, delta amplitude and SWS were only reduced with bilateral CFA injections and after 2 days of inflammation [41]. The same was also true for bilateral chronic constriction injury (CCI), a model of neuropathic pain, and skin incision, a model of surgical postoperative pain [41]. Furthermore, there are few reports on the interactions between photoperiod and pain, and they suggest that pain sensitivity can differ throughout the day, depending on species, strain, and type of noxious stimulus [42–44]. In general, the effects are highly situational and have not yet been assessed comprehensively. Our findings represent a comprehensive assessment in the context of how unilateral inflammatory pain affects sleep during periods of the day where an individual is more or less likely to the awake and active.

Our findings indicate that, in general, inflammatory pain promotes sleep prominently during the active phase, which then promotes disruption of normal daily activity patterns. Interestingly, a previous study showed that manipulations that disrupt or reduce sleep can potentiate thermal and mechanical hyperalgesia two weeks after CFA-induced inflammatory pain [19]. The same study characterized a neuronal ensemble in the nucleus accumbens (NAc), part of the limbic system and mesolimbic reward pathway, that exhibited increased activity with both nociceptive stimulation and wakefulness [19]. Activation of these neurons exacerbated nociceptive responses and reduced SWS in CCI mice but not those with sham surgeries, while inactivation had the opposite effect [19]. This ensemble primarily comprised dopamine type 1 receptor-expressing medium spiny neurons (D1-MSNs) projecting to the ventral tegmental area (VTA), which is known to regulate motivation as well as be part of the “pain matrix,” and to the preoptic area (POA) which is known to regulate sleep and wake [19]. These findings, when considered with reports that even brief periods of sleep disruption can be stressful as reflected by increased blood levels of corticosterone (CORT), suggest that sleep disruption interacts with stress to affect pain [38]. In the context of data from the present studies, one possibility is that CFA-induced increases in sleep represent adaptive responses that oppose the increases in pain sensitivity seen when sleep is reduced. Future work may determine if regulating specific sleep stages, either independently or in combination using approaches such as optogenetics and/or chemogenetics, at various time points during the day can alter the qualities of pain. Considering previous work establishing links among stress, sleep, and motivation, midbrain dopamine systems may be a target for manipulations that can affect the myriad signs of chronic pain syndromes [10, 11, 14, 18, 45, 46].

Another possibility is that CFA-induced pain alters systemic inflammation in ways that affect sleep. We previously reported that treatments that trigger inflammatory responses can produce persistent changes in sleep patterns, including reduced dark-phase activity and elevated SWS [47]. Indeed, studies in humans demonstrate complex interactions between inflammation, sleep, and mental health [48]. While it remains unclear how inflammatory factors can affect circuit function in brain areas that regulate motivation and emotion, it is now well-established that peripheral inflammation contributes importantly to psychiatric symptoms [49]. More studies are needed to determine the mechanisms by which inflammatory pain promotes sleep, which will enable the development of improved therapeutics. Clinically, inflammatory pain includes fibromyalgia and rheumatoid arthritis (RA). CFA-induced inflammatory pain closely emulates RA with some studies identifying rheumatoid factor in the joints of CFA-injected rodents [45, 50]. RA patients report longer sleep duration and more frequent daytime naps, and fatigue is highly correlated with individual ratings of pain [51]. Inflammation and sleep disturbances, particularly in RA, can have a reciprocal or feedforward relationship, often exacerbating pain sensitivity [52]. As RA becomes chronic, pain-associated fatigue and drowsiness diminished the quality of life. The present studies primarily focus on the first week after the induction of inflammatory pain, and future priorities include parsing whether these described effects on sleep architecture are due to pain, inflammation, or both. Future directions include characterizing how CFA affects inflammatory cytokine expression within neural circuits that regulate sleep and wakefulness, such as the preoptic area and locus coeruleus. Previous work has demonstrated that CFA promotes the expression of the cytokines IL-1β, TNF-α, and IL-6 in serum, whole brain, basolateral amygdala, and anterior cingulate [6, 21–25]. These molecules are regulated by sleep and circadian rhythms, and conversely, they regulate sleep and circadian rhythms in painful and inflammation-related conditions [21, 48]. Because we prioritized sleep endpoints and collected them continuously across the course of these experiments, we purposely minimized handling the mice to avoid additional stressors and disruptions of sleep. While we did not perform daily measurements body weight to minimize disruptions that will affect sleep, previous reports indicate that hindpaw injections of CFA do not produce changes in weight [53]. We acknowledge that changes in food intake related to chronic stress could affect sleep architecture, and this question could be assessed in future studies that span longer periods.

In addition to understanding how pain affects sleep, another area for investigation is whether baseline or subsequent individual differences in sleep traits contribute to the severity or persistence of inflammatory pain. In humans, sleep-related metrics are increasingly available via devices (e.g., smart phones, watches) that are highly prevalent [34]. Sleep patterns represent a potential biomarker that could be utilized in advance to predict responses to pain (via pre-surgical assessments), enabling proactive interventions that prevent, mitigate, or hasten recovery from pain. Moreover, as novel therapeutics are developed, leveraging sleep telemetry as a preclinical research endpoint will be vital to characterizing not only how new drugs affect disease symptoms, but also overall quality of life. Incorporating sleep as a translationally-relevant endpoint in animal models of pain is well-suited to explore the degree to which sleep patterns can be used to forecast behavioral responses to myriad forms of stress and challenges to homeostasis.

In summary, we found that inflammatory pain prominently increases sleep during normally active phases as well as transitions between sleep and wakefulness throughout the day. These effects align with clinical observations and establish a basis for mechanistic studies and use of these procedures to better predict outcomes in humans.

## Supplementary information

Supplemental Data

Statistics File

## Data Availability

Datasets are available upon request.
