# Supplementary material for: Inflammatory pain in mice induces light cycle-dependent effects on sleep architecture
Source: Neuropsychopharmacology. 2025 Jun 22;50(10):1595–605. doi: 10.1038/s41386-025-02152-w (PMC12340055; doi:10.1038/s41386-025-02152-w)
Supplement: Supplementary file 1 — Supplemental Data [file 41386_2025_2152_MOESM1_ESM.docx]

**Figure S1**. Representative periodograms, EEG signal, and EMG signal used for classifying awake, slow-wave sleep, and paradoxical or REM sleep in Neuroscore.

**Figure S2**. Mice were housed on automated piezoelectric sleep monitoring sensors. Sleep and wake behavior were recorded for 2 days of baseline, 7 days after saline injection, and 14 days after CFA injection. As % of average baseline (dotted line at 100%), (A) sleep duration, (B) sleep bout length (main effect of Time: F_(3.866,54.12)_=4.745, p=0.0026), and (C) wake bout length (main effect of Time: F_(5.981,83.73)_=3.265, p=0.0063) during lights-on. As % of average baseline (dotted line at 100%), (D) sleep duration (Time x Sex interaction: F(_11,154_)=10.28, p<0.0001), (E) sleep bout length (Time x Sex interaction F(_11,154_)=3.355, p=0.0004), and (F) wake bout length (main effect of Time: F_(4.914,68.80)_=6.140, p=0.0001; main effect of Sex F_(1,14)_=5.984, p=0.0283) during lights-off. Teal squares indicate males and magenta circles indicate females. * indicates significant main effect of time and sex interaction *** p<0.001, **** p<0.0001). ^ indicates significant main effect of time ^^ p<0.01, ^^^ p<0.001, ^^^^ p<0.0001). † indicates significant effect of sex († p<0.05).

**Figure S3**. Mice were implanted with wireless telemetry transmitters. EEG and EMG were recorded for 7 days of baseline, 7 days after isoflurane exposure, 7 days after saline injection, and 21 days after CFA injection. Mice were injected at the beginning of the light phase. As percent of average baseline (dotted line at 100%), (A) wake duration (F_(1.865,18.65)_=5.714, p=0.0129, Tukey’s ISO vs. CFA p=0.0346; Secondary analysis of the sexes individually: Time x Sex interaction F_(2,18)_=3.753, p=0.0434; Male vs. Female CFA: p=0.0377; Female ISO vs. CFA: p=0.0052; Female SAL vs. CFA: p=0.0493), (B) wake bout number (F_(1.515,15.15)_=0.7527, p=0.4529), and (C) locomotor activity (F_(1.919,19.19)_=1.925, p=0.1741; within-group CFA: t_10_=3.383, p=0.0070) during lights-on. As percent of average baseline (D) wake duration (F_(1.740,17.40)_=10.50, p=0.0014, ISO vs. CFA p=0.0063, SAL vs. CFA p=0.0018; within-group ISO: t_10_=2.346, p=0.0409; within-group SAL: t_10_=2.793, p=0.0.190; within-group CFA: t_10_=9.032, p<0.0001; Secondary analysis of the sexes individually: main effect of Time: F_(1.742,15.67)_=9.381, p=0.0028, Male SAL vs. CFA p=0.0360), (E) wake bout number (F_(1.735,17.35)_=6.000, p=0.0129, SAL vs. CFA p=0.0039; within-group ISO: t_10_=3.067, p=0.0119; within-group CFA: t_10_=4.569, p=0.0010) with secondary analysis (main effect of Time: F_(1.616,14.54)_=6.299, p=0.0142; Male SAL vs. CFA p=0.0240), and (F) locomotor activity (Friedman test F=16.91, p<0.0001; Dunn’s ISO vs. CFA p=0.0042, SAL vs. CFA p=0.0004; within-group Wilcoxon CFA p=0.0020; main effect of Time F_(1.251,11.26)_=7.316, p=0.0160; Female SAL vs. CFA p=0.0033) during lights-off. Teal squares indicate males and magenta circles indicate females. Ns indicates not significant. # indicates significant difference from theoretical baseline of 100% (# p<0.05, ## p<0.01, #### p<0.0001). * indicate significant group differences (* p<0.05, ** p<0.01, ***p<0.001).

**Figure S4**. Mice were implanted with wireless telemetry transmitters. EEG and EMG were recorded for 7 days of baseline, 7 days after isoflurane exposure, 7 days after saline injection, and 21 days after CFA injection. Mice were injected at the beginning of the light phase. As percent of average baseline (dotted line at 100%), (A) wake duration (main effect of Time: F_(4.253,34.28)_=3.907, p=0.0091), (B) REM duration, and (C) SWS duration during lights-on. As percent of average baseline (D) wake bouts (E) REM bouts (main effect of Time F_(3.796,30.59)_=4.104, p=0.0098), and (F) SWS bouts during lights-off. Teal squares indicate males and magenta circles indicate females. ^ indicates significant main effect of time (^^ p<0.01).

**Figure S5**. Mice were implanted with wireless telemetry transmitters. EEG and EMG were recorded for 7 days of baseline, 7 days after isoflurane exposure, 7 days after saline injection, and 21 days after CFA injection. Mice were injected at the beginning of the dark phase. As percent of average baseline (dotted line at 100%), (A) wake duration (F_(1.793,14.35)_=18.40, p=0.0001, ISO vs. CFA p=0.0004, SAL vs. CFA p=0.0072; within-group CFA: t_8_=11.28, p<0.0001), (B) wake bout number (F_(1.611,12.89)_=4.084, p=0.0493; within-group ISO: t_8_=2.413, p=0.0423; within-group CFA: t_8_=3.677, p=0.0062), and (C) locomotor activity (F_(1.344,10.75)_=3.582, p=0.0772) during lights-off. As percent of average baseline (D) wake duration(F_(1.839,14.72)_=5.328, p=0.0200, ISO vs. CFA p=0.0458; within-group CFA: t_8_=3.409, p=0.0092), (E) wake bout number (F_(1.895,15.16)_=0.05948, p=0.9352), and (F) locomotor activity (F_(1.809,14.47)_=1.824, p=0.1979) during lights-on. Teal squares indicate males and magenta circles indicate females. Ns indicates not significant. # indicates significant difference from theoretical baseline of 100% (# p<0.05, ## p<0.01, #### p<0.0001). * indicate significant group differences (* p<0.05, ** p<0.01, ***p<0.001).

**Figure S6**. Mice were implanted with wireless telemetry transmitters. EEG and EMG were recorded for 7 days of baseline, 7 days after isoflurane exposure, 7 days after saline injection, and 21 days after CFA injection. Mice were injected at the beginning of the dark phase. As percent of average baseline (dotted line at 100%), (A) wake duration (main effect of Time F_(5.052,35.36)_=6.133, p=0.0003; main effect of Sex F_(1,7)_=6.138, p=0.0424; Time x Sex interaction F_(17,119)_=2.538, p=0.0018), (B) REM duration (Time x Sex interaction F_(17,119)_=2.356, p=0.0038), and (C) SWS duration (Time x Sex interaction F_(17,119)_=2.486, p=0.0022) during lights-on. As percent of average baseline (D) wake bouts (main effect of Time F_(4.572,32.00)_=5.163, p=0.0018; main effect of Sex F_(1,7)_=9.711, p=0.0169), (E) REM bouts (main effect of Time F_(4.373,30.61)_=5.381, p=0.001), and (F) SWS bouts (Time x Sex interaction F_(17,119)_=2.0001, p=0.0162) during lights-off. Teal squares indicate males and magenta circles indicate females. # indicates significant difference from theoretical baseline of 100% (# p<0.05, ## p<0.01, #### p<0.0001). * indicates significant main effect of time and sex interaction (* p<0.05, ** p<0.01). ^ indicates significant main effect of time (^ p<0.05, ^^ p<0.01, ^^^^ p<0.0001). † indicates significant effect of sex († p<0.05, †† p<0.01, ††† p<0.001).

**Figure S7**. As percent of average baseline (dotted line at 100%), (A) temperature during lights-on for animals injected at the beginning of the light phase (F_(1.601,16.01)_=29.35, p<0.0001, ISO vs. CFA p<0.0001, SAL vs. CFA p=0.0024; within-group SAL: t_10_=4.477, p=0.0012; within-group CFA: t_10_=7.023, p<0.0001; main effect of Time F_(1.603,14.43)_=34.25, p<0.0001, Female SAL vs. CFA p=0.0165), (B) temperature during lights-off for animals injected at the beginning of the light phase (F_(1.932,19.32)_=35.72, p<0.0001, ISO vs. CFA p<0.0001, SAL vs. CFA p=0.0002; within-group ISO: t_10_=3.495, p=0.0058; within-group SAL: t_10_=6.000, p=0.0001; within-group CFA: t_10_=10.56, p<0.0001), (C) temperature during lights-on for animals injected at the beginning of the dark phase (F_(1.715,13.72)_=20.18, p=0.0001, ISO vs. CFA p=0.0001, SAL vs. CFA p=0.0144; within-group ISO t_8_=3.473, p=0.0084, SAL t_8_=6.292, p=0.0002, CFA t_8_=9.640, p<0.0001), (D) temperature during lights-off for animals injected at the beginning of the dark phase (F_(1.999,15.99)_=5.190, p=0.0183, p=0.0457; within-group ISO: t_8_=2.719, p=0.0263; within-group SAL: t_8_=5.111, p=0.0009; within-group CFA t_8_=2.959, p=0.0182) . Teal squares indicate males and magenta circles indicate females. # indicates significant difference from theoretical baseline of 100% (# p<0.05, ## p<0.01, ### p<0.001, #### p<0.0001). * indicate significant group differences (* p<0.05, ** p<0.01, *** p<0.001, **** p<0.0001).

| **Figure** | **Light Cycle** | **Method** | **Injections** | **Results: CFA:**  **Pooled Sexes:** | **Results: CFA:**  **Sex Differences:** |
| --- | --- | --- | --- | --- | --- |
| 2B – 2F | 12:12 L:D | Infrared beam breaks | 0900 hrs  ZT 3 | ↓ % variance  ↓ intradaily variability  ↓ period |  |
| 2G – 2K | 12:12 D:D | Infrared beam breaks | 0900 hrs  ZT 3 | ↑ relative amplitude  ↓ period |  |
| 3 | 12:12 L:D | piezoelectric sensors | 0900 hrs  ZT 3 | ↑ light and dark sleep duration  ↓ light sleep bout length  ↓ wake bout length  ↑ dark sleep bout length  ↓ dark wake bout length | Males  ↓ light wake bout length  Females  ↓ dark sleep bout length |
| 4  S3  S4 | 12:12 L:D | Wireless EEG | 0900 hrs  ZT 0 | ↓ light and dark wake duration  ↓ dark wake bouts  ↓ light and dark activity  ↑ dark REM duration & bouts  ↑ dark SWS duration & bouts | Females  ↓ wake light duration  ↓ dark activity  ↑ SWS light duration  Males  ↓ wake dark duration  ↓ wake dark bouts  ↑ REM dark bouts |
| 5  S5  S6 | 12:12 L:D | Wireless EEG | 0900 hrs  ZT 12 | ↓ light and dark wake duration  ↓ dark wake bouts  ↑ dark REM duration & bouts  ↑ light and dark SWS duration  ↑ light SWS bouts | Females  ↑ REM dark duration  ↑ SWS light & dark duration |

**Table 1**. Summary of CFA-specific effects for each figure, including light cycle (12 hours light, 12 hours dark or constant darkness), measurement method, injection time at zeitgeber (ZT) hour, for all mice pooled and for sex differences.
