# Supplementary material for: Inflammatory pain in mice induces light cycle-dependent effects on sleep architecture
Source: Neuropsychopharmacology. 2025 Jun 22;50(10):1595–605. doi: 10.1038/s41386-025-02152-w (PMC12340055; doi:10.1038/s41386-025-02152-w)
Supplement: Supplementary file 2 — Statistics File [file 41386_2025_2152_MOESM2_ESM.docx]

| **FIG 1: Paws** | **Repeated Measures 2-way ANOVA** | **Sidak’s, Left vs. Right** | **Sidak’s, vs. Baseline** |
| --- | --- | --- | --- |
| 1A  paw thickness | Time F_(5,55)_=138.5, p<0.0001  Paw F_(1,11)_=780.7, p<0.0001  Time x Paw F_(5,55)_=122.8, p<0.0001 | Baseline p=0.9026  24 hours post-SAL on left p=0.9995  24 hours post-CFA on right p<0.0001  1 week post-CFA on right p<0.0001  2 weeks post-CFA on right p<0.0001  3 weeks post-CFA on right p<0.0001 | Left  24 hours post-SAL on left p>0.9999  24 hours post-CFA on right p=0.9948  1 week post-CFA on right p>0.9999  2 weeks post-CFA on right p>0.9999  3 weeks post-CFA on right p=0.9996  Right  24 hours post-SAL on left p=0.4959  24 hours post-CFA on right p<0.0001  1 week post-CFA on right p<0.0001  2 weeks post-CFA on right p<0.0001  3 weeks post-CFA on right p<0.0001 |
| 1B  paw withdrawal | Time F_(5,55)_=26.51, p<0.0001  Paw F_(1,11)_=340.4, p<0.0001  Time x Paw F_(5,55)_=49.75, p<0.0001 | Baseline p=0.8902  24 hours post-SAL on left p=0.9995  24 hours post-CFA on right p<0.0001  1 week post-CFA on right p<0.0001  2 weeks post-CFA on right p<0.0001  3 weeks post-CFA on right p<0.0001 | Left  24 hours post-SAL on left p=0.9584  24 hours post-CFA on right p=0.3954  1 week post-CFA on right p=0.1572  2 weeks post-CFA on right p=0.1200  3 weeks post-CFA on right p=0.0424  Right  24 hours post-SAL on left p>0.9999  24 hours post-CFA on right p<0.0001  1 week post-CFA on right p<0.0001  2 weeks post-CFA on right p<0.0001  3 weeks post-CFA on right p<0.0001 |

| **FIG 2: Circadian** | **Paired T Test** | **1-sample T Test** | **2-Way ANOVA** | **Sidak’s Multiple Comparisons** |
| --- | --- | --- | --- | --- |
| 2B  Light/Dark  % Variance | t_10_=0.08684, p=0.9325 | SAL t_10_=3.420, p=0.0066  CFA t_10_=2.595, p=0.0267 | Time F_(1,9)_=4.561e-005, p=0.9948  Sex F_(1,9)_=0.1368, p=0.7200  Time x Sex F_(1,9)_=0.7481, p=0.4095 | SAL male vs. female p=0.7402  CFA male vs. female p=0.9992  SAL vs. CFA male p=0.7830  SAL vs. CFA female p=0.8198 |
| 2C  Light/Dark  Relative Amp | WM-PSR p=0.1016 | WSR SAL p=0.5195  WSR CFA p=0.7003 | Time F_(1,9)_=2.446, p=0.1523  Sex F_(1,9)_=0.7221, p=0.4175  Time x Sex F_(1,9)_=0.3586, p=0.5640 | SAL male vs. female p=0.5274  CFA male vs. female p=0.9138  SAL vs. CFA male p=0.7422  SAL vs. CFA female p=0.3229 |
| 2D  Light/Dark  Intradaily Variability | t_10_=2.094, p=0.0627 | SAL t_10_=1.639, p=0.1323  CFA t_10_=3.009, p=0.0131 | Time F_(1,9)_=3.885, p=0.0802  Sex F_(1,9)_=0.002251, p=0.9632  Time x Sex F_(1,9)_=0.5656, p=0.4712 | SAL male vs. female p=0.9564  CFA male vs. female p=0.9253  SAL vs. CFA male p=0.1429  SAL vs. CFA female p=0.6757 |
| 2E  Light/Dark  Period | WM-PSR p=0.9658 | WSR SAL p=0.0039  WSR CFA p=0.0273 | Time F_(1,9)_=0.2499, p=0.6291  Sex F_(1,9)_=1.060, p=0.3301  Time x Sex F_(1,9)_=0.2585, p=0.6234 | SAL male vs. female p=0.6563  CFA male vs. female p=0.4701  SAL vs. CFA male p=0.7230  SAL vs. CFA female p>0.9999 |
| 2F  Light/Dark  FFT Amplitude | t_10_=1.231, p=0.2466 | SAL t_10_=3.436, p=0.0064  CFA t_10_=0.07744, p=0.9398 | Time F_(1,9)_=1.284, p=0.2865  Sex F_(1,9)_=0.5101, p=0.4932  Time x Sex F_(1,9)_=0.8990, p=0.3678 | SAL male vs. female p=0.9921  CFA male vs. female p=0.4501  SAL vs. CFA male p=0.2895  SAL vs. CFA female p=0.9906 |
| 2G  Dark/Dark  % Variance | t_11_=1.404, p=0.1878 | SAL t_11_=2.015, p=0.0690  CFA t_11_=0.7561, p=0.4655 | Time F_(1,10)_=2.170, p=0.1715  Sex F_(1,10)_=1.780, p=0.2117  Time x Sex F_(1,10)_=2.103, p=0.1776 | SAL male vs. female p=0.7606  CFA male vs. female p=0.1689  SAL vs. CFA male p=0.1269  SAL vs. CFA female p=0.9998 |
| 2H  Dark/Dark  Relative Amp | t_11_=2.035, p=0.0667 | SAL t_11_=2.510, p=0.0290  CFA t_11_=2.954, p=0.0131 | Time F_(1,10)_=3.923, p=0.0758  Sex F_(1,10)_=0.1918, p=0.6708  Time x Sex F_(1,10)_=0.4195, p=0.5318 | SAL male vs. female p=0.9661  CFA male vs. female p=0.8000  SAL vs. CFA male p=0.6007  SAL vs. CFA female p=0.1769 |
| 2I  Dark/Dark  Intradaily Variability | t_11_=1.995, p=0.0715 | SAL t_11_=0.3502, p=0.7328  CFA t_11_=0.8966, p=0.3892 | Time F_(1,10)_=4.174, p=0.0683  Sex F_(1,10)_=0.5493, p=0.4756  Time x Sex F_(1,10)_=1.540, p=0.2430 | SAL male vs. female p=0.9383  CFA male vs. female p=0.4948  SAL vs. CFA male p=0.8262  SAL vs. CFA female p=0.0834 |
| 2J  Dark/Dark  Period | WM-PSR p=0.7695 | WSR SAL p=0.0005  WSR CFA p=0.0005 | Time F_(1,10)_=2.414, p=0.1513  Sex F_(1,10)_=2.558, p=0.1408  Time x Sex F_(1,10)_=2.641, p=0.1352 | SAL male vs. female p=0.9988  CFA male vs. female p=0.0655  SAL vs. CFA male p=0.0944  SAL vs. CFA female p=0.9985 |
| 2K  Dark/Dark  FFT Amplitude | t_11_=1.1844, p=0.0923 | SAL t_11_=1.515, p=0.1580  CFA t_11_=0.05886, p=0.9541 | Time F_(1,10)_=3.396, p=0.0951  Sex F_(1,10)_=1.207, p=0.2977  Time x Sex F_(1,10)_=0.9898, p=0.3433 | SAL male vs. female p=0.7903  CFA male vs. female p=0.3221  SAL vs. CFA male p=0.1399  SAL vs. CFA female p=0.8082 |

| **FIG 3: Piezo** | **Paired T Test** | **1-sample T Test** | **2-Way ANOVA (CFA)** | **Sidak’s Multiple Comparisons (CFA)** |
| --- | --- | --- | --- | --- |
| 3B  Light  Sleep Duration | t_15_=1.629, p=0.1242 | WSR SAL p<0.0001  WSR CFA p=00.0182 | Time F_(1,14)_=2.977, p=0.1064  Sex F_(1,14)_=0.1036, p=0.7523  Time x Sex F_(1,14)_=2.833, p=0.1145 | SAL male vs. female p=0.4759  CFA male vs. female p=0.8287  SAL vs. CFA male p=0.9995  SAL vs. CFA female p=0.0596 |
| 3C  Light  Sleep Bout Length | WM-PSR p=0.0013 | WSR SAL p=0.3225  WSR CFA p=0.0027 | Time F_(1,14)_=23.44, p=0.0003  Sex F_(1,14)_=0.3278, p=0.5760  Time x Sex F_(1,14)_=8.746, p=0.0104 | SAL male vs. female p=0.0823  CFA male vs. female p=0.4332  SAL vs. CFA male p=0.3663  SAL vs. CFA female p=0.0002 |
| 3D  Light  Wake Bout Length | WM-PSR p=0.0042 | WSR SAL p=0.0155  WSR CFA p<0.0001 | Time F_(1,14)_=8.750, p=0.0104  Sex F_(1,14)_=0.1930, p=0.66671  Time x Sex F_(1,14)_=0.1735, p=0.6833 | SAL male vs. female p=0.7892  CFA male vs. female p>0.9999  SAL vs. CFA male p=0.0624  SAL vs. CFA female p=0.1790 |
| 3E  Dark  Sleep Duration | WM-PSR p=0.0027 | WSR SAL p=0.0004  WSR CFA p<0.0001 | Time F_(1,14)_=16.97, p=0.0010  Sex F_(1,14)_=11.11, p=0.0049  Time x Sex F_(1,14)_=2.850, p=0.1135 | SAL male vs. female p=0.0018  CFA male vs. female p=0.0386  SAL vs. CFA male p=0.0021  SAL vs. CFA female p=0.2035 |
| 3F  Dark  Sleep Bout Length | t_15_=0.1013, p=0.9207 | SAL t_15_=4.071, p=0.0010  CFA t_15_=3.652, p=0.0024 | Time F_(1,14)_=0.01056, p=0.9196  Sex F_(1,14)_=0.06816, p=0.7978  Time x Sex F_(1,14)_=1.440, p=0.2500 | SAL male vs. female p=0.7294  CFA male vs. female p=0.5038  SAL vs. CFA male p=0.6063  SAL vs. CFA female p=0.6982 |
| 3G  Dark  Wake Bout Length | t_15_=5.745, p<0.0001 | SAL t_15_=4.485, p=0.0004  CFA t_15_=15.34, p<0.0001 | Time F_(1,14)_=42.22, p<0.0001  Sex F_(1,14)_=24.21, p=0.0002  Time x Sex F_(1,14)_=5.188, p=0.0390 | SAL male vs. female p<0.0001  CFA male vs. female p=0.0300  SAL vs. CFA male p<0.0001  SAL vs. CFA female p=0.0196 |

| **FIG S2:**  **Piezo**  **Longitudinal** | **Repeated Measures 2-Way ANOVA** | | |
| --- | --- | --- | --- |
|  | **SAL – CFA Week 1 – CFA Week 2** | **SAL – CFA Week 1** | **CFA Week 1 – CFA Week 2** |
| S2A  Light  Sleep Duration | Time F_(3.496,48.94)_=3.970, p=0.0097  Sex F_(1,14)_=0.07503, p=0.7881  Time x Sex F_(20,280)_=2.611, p=0.0003 | Time F_(2.503,35.04)_=5.224, p=0.0066  Sex F_(1,14)_=0.6260, p=0.4420  Time x Sex F_(13,182)_=2.462, p=0.0042 | Time F_(4.552,63.73)_=1.179, p=0.3291  Sex F_(1,14)_=0.1270, p=0.7269  Time x Sex F_(13,182)_=1.167, p=0.3070 |
| S2B  Light  Sleep Bout Length | Time F_(4.426,61.97)_=2.897, p=0.0249  Sex F_(1,14)_=0.8844, p=0.3629  Time x Sex F_(20,280)_=2.618, p=0.0003 | Time F_(4.483,62.76)_=3.828, p=0.0058  Sex F_(1,14)_=0.08774, p=0.7714  Time x Sex F_(13,182)_=2.712, p=0.0016 | Time F_(3.866,54.12)_=4.745, p=0.0026  Sex F_(1,14)_=2.638, p=0.1267  Time x Sex F_(13,182)_=0.5303, p=0.9038 |
| S2C  Light  Wake Bout Length | Time F_(7.141,99.98)_=5.100, p<0.0001  Sex F_(1,14)_=2.627, p=0.1274  Time x Sex F_(20,280)_=1.310, p=0.1709 | Time F_(5.693,79.70)_=6.511, p<0.0001  Sex F_(1,14)_=4.782, p=0.0462  Time x Sex F_(13,182)_=1.246, p=0.2505 | Time F_(5.981,83.73)_=3.265, p=0.0063  Sex F_(1,14)_=0.8160, p=0.3816  Time x Sex F_(13,182)_=1.238, p=0.2559 |
| S2D  Dark  Sleep Duration | Time F_(1.919,26.86)_=15.04, p<0.0001  Sex F_(1,14)_=11.24, p=0.0047  Time x Sex F_(18,252)_=12.89, p<0.0001 | Time F_(2.316,32.43)_=15.47, p<0.0001  Sex F_(1,14)_=13.60, p=0.0024  Time x Sex F_(12,168)_=13.70, p<0.0001 | Time F_(1.728,24.19)_=20.83, p<0.0001  Sex F_(1,14)_=7.393, p=0.0166  Time x Sex F_(11,154)_=10.28, p<0.0001 |
| S2E  Dark  Sleep Bout Length | Time F_(4.876,68.26)_=4.818, p=0.0009  Sex F_(1,14)_=7.712, p<0.0001  Time x Sex F_(18,252)_=4.789, p<0.0001 | Time F_(4.081,57.14)_=2.580, p=0.0457  Sex F_(1,14)_=0.9186, p=0.3541  Time x Sex F_(12,168)_=4.785, p<0.0001 | Time F_(3.835,53.68)_=5.230, p=0.0014  Sex F_(1,14)_=0.2345, p=0.6357  Time x Sex F_(11,154)_=3.355, p=0.0004 |
| S2F  Dark  Wake Bout Length | Time F_(2.898,40.57)_=4.756, p=0.0067  Sex F_(1,14)_=9.025, p=0.0095  Time x Sex F_(18,252)_=3.495, p<0.0001 | Time F_(4.692,65.69)_=5.718, p=0.0003  Sex F_(1,14)_=15.25, p=0.0016  Time x Sex F_(12,168)_=4.110, p<0.0001 | Time F_(4.914,68.80)_=6.140, p=0.0001  Sex F_(1,14)_=5.984, p=0.0283  Time x Sex F_(11,154)_=1.604, p=0.1026 |

| **FIG S3:**  **Light-Onset Wake EEG** | **RM 1-Way ANOVA** | **Multiple Comparisons** | **1-sample T-Test** | **2-Way ANOVA** | **Sidak’s Multiple Comparisons** |
| --- | --- | --- | --- | --- | --- |
| S3A  Light Duration | F_(1.865,18.65)_=5.714, p=0.0129 | ISO vs. CFA p=0.0346  ISO vs. SAL p=0.4464  SAL vs. CFA p=0.0951 | ISO t_10_=1.755, p=0.1097  SAL t_10_=0.7235, p=0.4860  CFA t_10_=1.835, p=0.0964 | Time F_(1.868,16.81)_=6.316, p=0.0100  Sex F_(1,9)_=0.3828, p=0.5514  Time x Sex F_(2,18)_=3.753, p=0.0434 | male vs. female:  ISO p=0.9546  SAL p=0.9994  CFA p=0.0377  male:  ISO vs. SAL p=0.9546  ISO vs. CFA p=0.8846  SAL vs. CFA p=0.9644  female:  ISO vs. SAL p=0.3106  ISO vs. CFA p=0.0052  SAL vs. CFA p=0.0493 |
| S3B  Light Bout | F_(1.515,15.15)_=0.7527, p=0.4529 | ISO vs. CFA p=0.9819  ISO vs. SAL p=0.7095  SAL vs. CFA p=0.5035 | ISO t_10_=1.158, p=0.2736  SAL t_10_=2.142, p=0.0579  CFA t_10_=1.070, p=0.3097 | Time F_(1.588,14.29)_=0.9882, p=0.3777  Sex F_(1,9)_=0.1001, p=0.7590  Time x Sex F_(2,18)_=1.500, p=0.2496 | male vs. female:  ISO p=0.8898  SAL p=0.9413  CFA p=0.9424  male:  ISO vs. SAL p=0.2624  ISO vs. CFA p=0.9748  SAL vs. CFA p=0.2742  female:  ISO vs. SAL p=0.9695  ISO vs. CFA p=0.9703 SAL vs. CFA p=0.9953 |
| S3C  Light Activity | F_(1.919,19.19)_=1.925, p=0.1741 | ISO vs. CFA p=0.8484  ISO vs. SAL p=0.4344  SAL vs. CFA p=0.1306 | ISO t_10_=1.518, p=0.1600  SAL t_10_=1.810, p=0.1005  CFA t_10_=3.383, p=0.0070 | Time F_(1.987,17.89)_=1.968, p=0.1690  Sex F_(1,9)_=1.466, p=0.2568  Time x Sex F_(2,18)_=1.419, p=0.2677 | male vs. female:  ISO p=0.2539  SAL p=0.9993  CFA p=0.9886  male:  ISO vs. SAL p=0.0663  ISO vs. CFA p=0.9910  SAL vs. CFA p=0.0052  female:  ISO vs. SAL p=0.8924  ISO vs. CFA p=0.3420 SAL vs. CFA p=0.5903 |
| S3D  Dark Duration | F_(1.740,17.40)_=10.50, p=0.0014 | ISO vs. CFA p=0.0063  ISO vs. SAL p=0.9874  SAL vs. CFA p=0.0018 | ISO t_10_=2.346, p=0.0409  SAL t_10_=2.793, p=0.0.190  CFA t_10_=9.032, p<0.0001 | Time F_(1.742,15.67)_=9.381, p=0.0028  Sex F_(1,9)_=1.109, p=0.3198  Time x Sex F_(2,18)_=0.01986, p=0.9804 | male vs. female:  ISO p=0.9776  SAL p=0.7876  CFA p=0.9058  male:  ISO vs. SAL p=0.9997  ISO vs. CFA p=0.1317  SAL vs. CFA p=0.0360  female:  ISO vs. SAL p=0.9677  ISO vs. CFA p=0.0777  SAL vs. CFA p=0.0652 |
| S3E  Dark Bout | F_(1.735,17.35)_=6.000, p=0.0129 | ISO vs. CFA p=0.3694  ISO vs. SAL p=0.2368  SAL vs. CFA p=0.0039 | ISO t_10_=3.067, p=0.0119  SAL t_10_=0.9989, p=0.3414  CFA t_10_=4.569, p=0.0010 | Time F_(1.616,14.54)_=6.299, p=0.0142  Sex F_(1,9)_=0.6718, p=0.4336  Time x Sex F_(2,18)_=0.8924, p=0.4271 | male vs. female:  ISO p=0.9595  SAL p=0.9990  CFA p=0.3876  male:  ISO vs. SAL p=0.3572  ISO vs. CFA p=0.0987  SAL vs. CFA p=0.0240  female:  ISO vs. SAL p=0.7756  ISO vs. CFA p=0.8428  SAL vs. CFA p=0.1414 |
| S3F  Dark Activity | Friedman=16.91, p<0.0001 | D’s ISO vs. CFA p=0.0042  D’s ISO vs. SAL p>0.9999  D’s SAL vs. CFA p=0.0004 | WSR ISO p=0.8311  WSR SAL p=0.5195  WSR CFA p=0.0020 | Time F_(1.251,11.26)_=7.316, p=0.0160  Sex F_(1,9)_=1.111, p=0.3193  Time x Sex F_(2,18)_=0.09453, p=0.9102 | male vs. female:  ISO p=0.9192  SAL p=0.8727  CFA p=0.5536  male:  ISO vs. SAL p=0.8874  ISO vs. CFA p=0.1103  SAL vs. CFA p=0.1480  female:  ISO vs. SAL p=0.9989  ISO vs. CFA p=0.1254  SAL vs. CFA p=0.0033 |

| **FIG 4:**  **Light-Onset Sleep EEG** | **RM 1-Way ANOVA** | **Multiple Comparisons** | **1-sample T-Test** | **2-Way ANOVA** | **Sidak’s Multiple Comparisons** |
| --- | --- | --- | --- | --- | --- |
| 4B  Light  REM Duration | F_(1.986,19.86)_=0.02847, p=0.9714 | ISO vs. SAL p=0.2602  ISO vs. CFA p=0.9728  SAL vs. CFA p=0.9996 | ISO t_10_=0.2918, p=0.7764  SAL t_10_=0.02679, p=0.9792  CFA t_10_=0.0002580, p=0.9998 | Time F_(1.992,17.93)_=0.01918, p=0.9808  Sex F_(1,9)_=0.9225, p=0.3619  Time x Sex F_(2,18)_=0.2536, p=0.7787 | male vs. female:  ISO p=0.9256  SAL p=0.5999  CFA p=0.9247  male:  ISO vs. SAL p=0.8309  ISO vs. CFA p=0.9998  SAL vs. CFA p=0.9435  female:  ISO vs. SAL p=0.8855  ISO vs. CFA p=0.9718  SAL vs. CFA p=0.9035 |
| 4C  Light  REM Bouts | F_(1.427,14.27)_=0.745,p=0.4489 | ISO vs. SAL p=0.7055  ISO vs. CFA p=0.9873  SAL vs. CFA p=0.5424 | ISO t_10_=1.900, p=0.0867  SAL t_10_=1.784, p=0.1048  CFA t_10_=1.600, p=0.1406 | Time F_(1.426,12.83)_=0.6753, p=0.4777  Sex F_(1,9)_=2.119, p=0.1794  Time x Sex F_(2,18)_=0.01650, p=0.9836 | male vs. female:  ISO p=0.2635  SAL p=0.8442  CFA p=0.4972  male:  ISO vs. SAL p=0.2290  ISO vs. CFA p=0.9170  SAL vs. CFA p=0.2600  female:  ISO vs. SAL p=0.9298  ISO vs. CFA p=0.9787  SAL vs. CFA p=0.8349 |
| 4D  Light  SWS Duration | F_(1.390,13.90)_=4.341, p=0.0457 | ISO vs. SAL p=0.1879  ISO vs. CFA p=0.0901  SAL vs. CFA p=0.2641 | ISO t_10_=1.541, p=0.1543  SAL t_10_=0.2169, p=0.8327  CFA t_10_=1.663, p=0.1273 | Time F_(1.639,14.75)_=5.500, p=0.0207  Sex F_(1,9)_=0.001246, p=0.9726  Time x Sex F_(2,18)_=6.197, p=0.0090 | male vs. female:  ISO p=0.6517  SAL p=0.7258  CFA p=0.2009  male:  ISO vs. SAL p=0.6178  ISO vs. CFA p=0.9977  SAL vs. CFA p=0.7716  female:  ISO vs. SAL p=0.3483  ISO vs. CFA p=0.0133  SAL vs. CFA p=0.0302 |
| 4E  Light  SWS Bouts | F_(1.960,19.60)_=0.8844, p=0.4269 | ISO vs. SAL p=0.4759  ISO vs. CFA p=0.5411  SAL vs. CFA p=0.9694 | ISO t_10_=0.6498, p=0.5305  SAL t_10_=0.9533, p=0.3629  CFA t_10_=0.7360, p=0.4786 | Time F_(1.945,17.50)_=0.8065, p=0.4592  Sex F_(1,9)_=0.3365, p=0.5761  Time x Sex F_(2,18)_=0.3991, p=0.6767 | male vs. female:  ISO p=0.3838  SAL p=0.8767  CFA p=0.9988  male:  ISO vs. SAL p=0.4337  ISO vs. CFA p=0.9556  SAL vs. CFA p=0.6705  female:  ISO vs. SAL p=0.8103  ISO vs. CFA p=0.5864  SAL vs. CFA p=0.9394 |
| 4G  Dark  REM Duration | F_(1.446,13.01)_=10.72, p=0.0032 | ISO vs. SAL p=0.9801  ISO vs. CFA p=0.0224  SAL vs. CFA p=0.0115 | ISO t_9_=1.794, p=0.1064  SAL t_9_=0.9019, p=0.3906  CFA t_9_=3.462, p=0.0071 | Time F_(1.443,11.55)_=9.395, p=0.0062  Sex F_(1,8)_=0.07773, p=0.7875  Time x Sex F_(2,16)_=0.03706, p=0.9637 | male vs. female:  ISO p=0.9518  SAL p=0.9444  CFA p>0.9999  male:  ISO vs. SAL p=0.7519  ISO vs. CFA p=0.3416  SAL vs. CFA p=0.2465  female:  ISO vs. SAL p>0.9999  ISO vs. CFA p=0.0835  SAL vs. CFA p=0.0676 |
| 4H  Dark  REM Bouts | F_(1.716,17.16)_=6.088, p=0.0126 | ISO vs. SAL p=0.8135  ISO vs. CFA p=0.1058  SAL vs. CFA p=0.0092 | ISO t_10_=2.740, p=0.0208  SAL t_10_=1.653, p=0.1293  CFA t_10_=3.455, p=0.0062 | Time F_(1.489,13.40)_=6.893, p=0.0130  Sex F_(1,9)_=0.5470, p=0.4784  Time x Sex F_(2,18)_=1.511, p=0.2473 | male vs. female:  ISO p=0.8254  SAL p>0.9999  CFA p=0.6707  male:  ISO vs. SAL p=0.5456  ISO vs. CFA p=0.2766  SAL vs. CFA p=0.0443  female:  ISO vs. SAL p=0.9924  ISO vs. CFA p=0.4564  SAL vs. CFA p=0.0962 |
| 4I  Dark  SWS Duration | F_(1.976,28.66)_=8.823, p=0.0011 | ISO vs. SAL p=0.9696  ISO vs. CFA p=0.0112  SAL vs. CFA p=0.0170 | ISO t_9_=2.088, p=0.0676  SAL t_10_=3.188, p=0.0097  CFA t_10_=6.548, p<0.0001 | Time F_(1.937,25.18)_=8.024, p=0.0022  Sex F_(1,26)_=0.2294, p=0.6360  Time x Sex F_(2,26)_=0.6259, p=0.5427 | male vs. female:  ISO p=0.8492  SAL p=0.8013  CFA p=0.9290  male:  ISO vs. SAL p=0.9701  ISO vs. CFA p=0.3111  SAL vs. CFA p=0.2992  female:  ISO vs. SAL p=0.9988  ISO vs. CFA p=0.0862  SAL vs. CFA p=0.0699 |
| 4J  Dark  SWS Bouts | Friedman=6.545, p=0.0435 | D’s ISO vs. SAL p=0.6025  D’s ISO vs. CFA p=0.6025  D’s SAL vs. CFA p=0.0315 | WSR ISO p=0.2061  WSR SAL p=0.4648  WSR CFA p=0.0010 | Time F_(1.340,12.06)_=2.151, p=0.1663  Sex F_(1,9)_=1.053, p=0.3317  Time x Sex F_(2,18)_=1.061, p=0.3668 | male vs. female:  ISO p=0.5052  SAL p=0.9749  CFA p=0.8165  male:  ISO vs. SAL p=0.9743  ISO vs. CFA p=0.6289  SAL vs. CFA p=0.1943  female:  ISO vs. SAL p=0.2423  ISO vs. CFA p=0.9992  SAL vs. CFA p=0.0526 |

| **FIG S4:**  **Light-Onset**  **Dark-Phase**  **Longitudinal** | **Repeated Measures 2-Way ANOVA**  **Mixed-Effects Analysis** | | |
| --- | --- | --- | --- |
|  | **ISO – SAL – CFA Weeks 1-3** | **ISO – SAL – CFA Week 1** | **CFA Weeks 1-3** |
| S4A  Awake Duration | Time F_(5.306,44.83)_=3.298, p=0.0114  Sex F_(1.9)_=0.5540, p=0.4757  Time x Sex F_(29.245)_=0.7131, p=0.8617 | Time F_(5.559,50.03)_=5.863, p=0.0002  Sex F_(1.9)_=0.2301, p=0.6429  Time x Sex F_(17,153)_=0.6080, p=0.8821 | Time F_(4.253,34.28)_=3.907, p=0.0091  Sex F_(1,9)_=0.7323, p=0.4143  Time x Sex F_(17,137)_=0.9321, p=0.5382 |
| S4B=  REM Duration | Time F_(4.029,33.62)_=1.729, p=0.1664  Sex F_(1,9)_=0.01789, p=0.8965  Time x Sex F_(29.242)_=0.7619, p=0.8075 | Time F_(3.127,27.77)_=1.812, p=0.1663  Sex F_(1,9)_=0.1012, p=0.7577  Time x Sex F_(17,151)_=0.7730, p=0.7216 | Time F_(3.285,26.09)_=2.413, p=0.0847  Sex F_(1,9)_=0.2456, p=0.6320  Time x Sex F_(17,135)_=0.4112, p=0.9810 |
| S4C  SWS Duration | Time F_(3.865,32.65)_=2.095, p=0.1059  Sex F_(1,9)_=0.07227  Time x Sex F_(29,245)_=0.6631, p=0.9073 | Time F_(4.616,41.55)_=3.763, p=0.0079  Sex F_(1,9)_=5.547e-005, p=0.9942  Time x Sex F_(17.153)_=0.6748, p=0.8240 | Time F_(3.326,26.80)_=2.154, p=0.1116  Sex F_(1,9)_=0.2133, p=0.6552  Time x Sex F_(17,137)_=0.8135, p=0.6754 |
| S4D  Awake Bouts | Time F_(3.245,27.42)_=2.743, p=0.0585  Sex F_(1.9)_=0.2900  Time x Sex F_(29,245)_=0.9056 | Time F_(4.167,37.50)_=4.718, p=0.0031  Sex F_(1,9)_=0.1474, p=0.7099  Time x Sex F_(17,153)_=0.8460 | Time F_(2.437,19.64)_=2.871, p=0.0718  Sex F_(1,9)_=0.6151, p=0.4530  Time x Sex F_(17,136)_=0.8246, p=0.6626 |
| S4E  REM Bouts | Time F_(4.467,37.74)_=3.275, p=0.0179  Sex F_(1,9)_=0.1999, p=0.6654  Time x Sex F_(29,.245)_=1.197 | Time F_(4.237,38.13)_=4.589, p=0.0035  Sex F_(1.9)_=0.1216, p=0.7353  Time x Sex F_(17,153)_=1.577, p=0.0766 | Time F_(3.796,30.59)_=4.104, p=0.0098  Sex F_(1,9)_=0.4545, p=0.5171  Time x Sex F_(17,137)_=0.7882, p=0.7041 |
| S4F  SWS Bouts | Time F_(2.961,25.02)_=1.413, p=0.2626  Sex F_(1,9)_=0.1440, p=0.7131  Time x Sex F_(29,245)_=1.148, p=0.2817 | Time F_(1.925,17.32)_=1.487, p=0.2533  Sex F_(1,9)_=0.4655, p=0.5123  Time x Sex F_(17,153)_=0.9112, p=0.5620 | Time F_(2.598,20.94)_=1.524, p=0.2396  Sex F_(1,9)_=0.3764, p=0.5547  Time x Sex F_(17,137)_=1.222, p=0.2553 |

| **FIG S5:**  **Dark-Onset Wake EEG** | **RM 1-Way ANOVA** | **Multiple Comparisons** | **1-sample T-Test** | **2-Way ANOVA** | **Sidak’s Multiple Comparisons** |
| --- | --- | --- | --- | --- | --- |
| S5A  Dark Duration | F_(1.793,14.35)_=18.40, p=0.0001 | ISO vs. CFA p=0.0004  ISO vs. SAL p=0.7298  SAL vs. CFA p=0.0072 | ISO t_8_=0.2799, p=0.7866  SAL t_8_=0.5410, p=0.6032  CFA t_8_=11.28, p<0.0001 | Time F_(1.685,11.79)_=16.89, p=0.0005  Sex F_(1,7)_=4.960, p=0.0612  Time x Sex F_(2,14)_=0.7989, p=0.4693 | male vs. female:  ISO p=0.1459  SAL p=0.6021  CFA p=0.9455  male:  ISO vs. SAL p=0.9739  ISO vs. CFA p=0.0277  SAL vs. CFA p=0.1769  female:  ISO vs. SAL p=0.6296  ISO vs. CFA p=0.0124  SAL vs. CFA p=0.0606 |
| S5B  Dark Bout | F_(1.611,12.89)_=4.084, p=0.0493 | ISO vs. CFA p=0.0748  ISO vs. SAL p=0.6731  SAL vs. CFA p=0.0821 | ISO t_8_=2.413, p=0.0423  SAL t_8_=0.7830, p=0.4562  CFA t_8_=3.677, p=0.0062 | Time F_(1.295,9.062)_=4.607, p=0.0533  Sex F_(1,7)_=1.272, p=0.2965  Time x Sex F_(2,14)_=1.264, p=0.3130 | male vs. female:  ISO p=0.9970  SAL p=0.9532  CFA p=0.2159  male:  ISO vs. SAL p=0.9273  ISO vs. CFA p=0.0898  SAL vs. CFA p=0.0778  female:  ISO vs. SAL p=0.7555  ISO vs. CFA p=0.4432  SAL vs. CFA p=0.6028 |
| S5C  Dark Activity | F_(1.344,10.75)_=3.582, p=0.0772 | ISO vs. CFA p=0.1166  ISO vs. SAL p=0.9847  SAL vs. CFA p=0.2101 | ISO t_8_=0.9787, p=0.3564  SAL t_8_=1.113, p=0.2981  CFA t_8_=1.593, p=0.1499 | Time F_(1.322,9.255)_=3.122, p=0.1039  Sex F_(1,7)_=2.618, p=0.1497  Time x Sex F_(2,14)_=0.08173, p=0.9220 | male vs. female:  ISO p=0.5180  SAL p=0.7989  CFA p=0.8546  male:  ISO vs. SAL p=0.9230  ISO vs. CFA p=0.5696  SAL vs. CFA p=0.7445  female:  ISO vs. SAL p=0.8542  ISO vs. CFA p=0.1013  SAL vs. CFA p=0.1709 |
| S5D  Light Duration | F_(1.839,14.72)_=5.328, p=0.0200 | ISO vs. CFA p=0.0458  ISO vs. SAL p=0.9458  SAL vs. CFA p=0.6344 | ISO t_8_=1.052, p=0.3236  SAL t_8_=1.784, p=0.1122  CFA t_8_=3.409, p=0.0092 | Time F_(1.924,13.47)_=5.637, p=0.0174  Sex F_(1,7)_=0.4807, p=0.5104  Time x Sex F_(2,14)_=0.9554, p=0.4084 | male vs. female:  ISO p=0.6294  SAL p=0.8048  CFA p=0.9266  male:  ISO vs. SAL p=0.2029  ISO vs. CFA p=0.2440  SAL vs. CFA p=0.5076  female:  ISO vs. SAL p=0.3752  ISO vs. CFA p=0.1225  SAL vs. CFA p=0.9994 |
| S5E  Light Bout | F_(1.895,15.16)_=0.05948, p=0.9352 | ISO vs. CFA p=0.9978  ISO vs. SAL p=0.9270  SAL vs. CFA p=0.9681 | ISO t_8_=007404, p=0.9943  SAL t_8_=0.3421, p=0.7411  CFA t_8_=0.06136, p=0.9526 | Time F_(1.888,13.22)_=0.06222, p=0.9321  Sex F_(1,7)_=0.3217, p=0.5883  Time x Sex F_(2,14)_=0.1430, p=0.8680 | male vs. female:  ISO p=0.9989  SAL p=0.9317  CFA p=0.8801  male:  ISO vs. SAL p=0.5320  ISO vs. CFA p=0.9486  SAL vs. CFA p=0.9988  female:  ISO vs. SAL p=0.999  ISO vs. CFA p=0.8994  SAL vs. CFA p=0.9614 |
| S5F  Light Activity | F_(1.809,14.47)_=1.824, p=0.1979 | ISO vs. CFA p=0.9495  ISO vs. SAL p=0.3911  SAL vs. CFA p=0.2783 | ISO t_8_=0.1740, p=0.8662  SAL t_8_=1.664, p=0.1347  CFA t_8_=0.4916, p=0.6362 | Time F_(1.840,12.88)_=1.507, p=0.2571  Sex F_(1,7)_=0.2445, p=0.6361  Time x Sex F_(2,14)_=0.4105, p=0.6711 | male vs. female:  ISO p=0.9943  SAL p=0.7679  CFA p=0.9985  male:  ISO vs. SAL p=0.9379  ISO vs. CFA p=0.9211  SAL vs. CFA p=0.8274  female:  ISO vs. SAL p=0.4145  ISO vs. CFA p>0.9999  SAL vs. CFA p=0.3392 |

| **FIG 5:**  **Light-Onset Sleep EEG** | **RM 1-Way ANOVA** | **Multiple Comparisons** | **1-sample T-Test** | **2-Way ANOVA** | **Sidak’s Multiple Comparisons** |
| --- | --- | --- | --- | --- | --- |
| 5B  Dark  REM Duration | F_(1.736,13.89)_=6.291, p=0.0136 | ISO vs. SAL p=0.8926  ISO vs. CFA p=0.0313  SAL vs. CFA p=0.0575 | ISO t_8_=1.677, p=0.1320  SAL t_8_=1.271, p=0.2393  CFA t_8_=3.466, p=0.0085 | Time F_(1.558,10.91)_=8.791, p=0.0075  Sex F_(1,7)_=6.058, p=0.0434  Time x Sex F_(2,14)_=2.929, p=0.0866 | male vs. female:  ISO p=0.9088  SAL p=0.7928  CFA p=0.1226  male:  ISO vs. SAL p=0.9844  ISO vs. CFA p=0.0737  SAL vs. CFA p=0.1533  female:  ISO vs. SAL p=0.8469  ISO vs. CFA p=0.0245  SAL vs. CFA p=0.3404 |
| 5C  Dark  REM Bouts | F_(1.671,13.37)_=2.908, p=0.0961 | ISO vs. SAL p=0.4738  ISO vs. CFA p=0.3455  SAL vs. CFA p=0.1731 | ISO t_8_=2.429, p=0.0413  SAL t_8_=0.8644, p=0.4125  CFA t_8_=2.794, p=0.0234 | Time F_(1.623,11.36)_=4.840, p=0.0357  Sex F_(1,7)_=1.319, p=0.2885  Time x Sex F_(2,14)_=4.040, p=0.0412 | male vs. female:  ISO p=0.9811  SAL p=0.9989  CFA p=0.1086  male:  ISO vs. SAL p=0.5201  ISO vs. CFA p=0.1137  SAL vs. CFA p=0.1370  female:  ISO vs. SAL p=0.8112  ISO vs. CFA p=0.7569  SAL vs. CFA p=0.9093 |
| 5D  Dark  SWS Duration | F_(1.571,12.57)_=15.37, p=0.0007 | ISO vs. SAL p=0.6545  ISO vs. CFA p=0.0002  SAL vs. CFA p=0.0127 | ISO t_8_=0.3528, p=0.7334  SAL t_8_=0.7649, p=0.4663  CFA t_8_=9.234, p<0.0001 | Time F_(1.402,9.816)_=13.83, p=0.0025  Sex F_(1,7)_=3.202, p=0.1167  Time x Sex F_(2,14)_=0.6392, p=0.5424 | male vs. female:  ISO p=0.0639  SAL p=0.7748  CFA p=0.9884  male:  ISO vs. SAL p=0.9421  ISO vs. CFA p=0.0652  SAL vs. CFA p=0.2839  female:  ISO vs. SAL p=0.6475  ISO vs. CFA p=0.0020  SAL vs. CFA p=0.0646 |
| 5E  Dark  SWS Bouts | F_(1.683,13.47)_=5.901, p=0.0177 | ISO vs. SAL p=0.9051  ISO vs. CFA p=0.0305  SAL vs. CFA p=0.0225 | ISO t_8_=0.1854, p=0.8576  SAL t_8_=0.2811, p=0.7858  CFA t_8_=7.063, p=0.0001 | Time F_(1.712,11.99)_=5.198, p=0.0274  Sex F_(1,7)_=0.8850, p=0.3782  Time x Sex F_(2,14)_=0.4156, p=0.6678 | male vs. female:  ISO p=0.5981  SAL p=0.9779  CFA p=0.7381  male:  ISO vs. SAL p=0.9737  ISO vs. CFA p=0.2695  SAL vs. CFA p=0.1226  female:  ISO vs. SAL p=0.7412  ISO vs. CFA p=0.1364  SAL vs. CFA p=0.2159 |
| 5G  Light  REM Duration | F_(1.240,9.919)_=1.053, p=0.3478 | ISO vs. SAL p=0.9540  ISO vs. CFA p=0.5826  SAL vs. CFA p=0.3025 | ISO t_8_=0.5991, p=0.5657  SAL t_8_=0.7489, p=0.4753  CFA t_8_=0.9799, p=0.3558 | Time F_(1.315,9.204)_=1.013, p=0.3651  Sex F_(1,7)_=0.004537, p=0.9482  Time x Sex F_(2,14)_=3.529, p=0.0574 | male vs. female:  ISO p=0.6958  SAL p=0.9219  CFA p=0.3485  male:  ISO vs. SAL p=0.1300  ISO vs. CFA p=0.7846  SAL vs. CFA p=0.9470  female:  ISO vs. SAL p=0.3729  ISO vs. CFA p=0.1999  SAL vs. CFA p=0.1164 |
| 5H  Light  REM Bouts | F_(1.826,14.61)_=0.259, p=0.7555 | ISO vs. SAL p=0.7005  ISO vs. CFA p=0.9849  SAL vs. CFA p=0.8874 | ISO t_8_=0.01654, p=0.9872  SAL t_8_=0.6997, p=0.5039  CFA t_8_=0.2143, p=0.8357 | Time F_(1.874,13.12)_=0.2681, p=0.7552  Sex F_(1,7)_=0.04685  Time x Sex F_(2,14)_=1.217, p=0.3255 | male vs. female:  ISO p=0.9542  SAL p=0.9993  CFA p=0.5681  male:  ISO vs. SAL p=0.3269  ISO vs. CFA p=0.6373  SAL vs. CFA p=0.8664  female:  ISO vs. SAL p=0.9596  ISO vs. CFA p=0.4862  SAL vs. CFA p=0.6295 |
| 5I  Light  SWS Duration | F_(1.948,15.58)_=8.956, p=0.0027 | ISO vs. SAL p=0.0865  ISO vs. CFA p=0.0062  SAL vs. CFA p=0.2479 | ISO t_8_=1.750, p=0.1182  SAL t_8_=1.180, p=0.2717  CFA t_8_=4.117, p=0.0034 | Time F_(1.928,13.50)_=8.119, p=0.0051  Sex F_(1,7)_=1.050, p=0.3396  Time x Sex F_(2,14)_=0.4464, p=0.6487 | male vs. female:  ISO p=0.9247  SAL p=0.6674  CFA p=0.9178  male:  ISO vs. SAL p=0.4970  ISO vs. CFA p=0.2848  SAL vs. CFA p=0.2746  female:  ISO vs. SAL p=0.1944  ISO vs. CFA p=0.0149  SAL vs. CFA p=0.7552 |
| 5I  Light SWS Bouts | F_(1.619, 12.96)_=6.095, p=0.0175 | ISO vs. SAL p=0.0665  ISO vs. CFA p=0.4917  SAL vs. CFA p=0.0193 | ISO t_8_=0.09420, p=0.9273  SAL t_8_=2.022, p=0.0779  CFA t_8_=1.852, p=0.1011 | Time F_(1.578,11.04)_=5.596, p=0.0261  Sex F_(1,7)_=0.2209, p=0.6527  Time x Sex F_(2,14)_=0.3548, p=0.7075 | male vs. female:  ISO p=0.9981  SAL p=0.9216  CFA p=0.9127  male:  ISO vs. SAL p=0.0451  ISO vs. CFA p=0.9238  SAL vs. CFA p=0.3166  female:  ISO vs. SAL p=0.5798  ISO vs. CFA p=0.5508  SAL vs. CFA p=0.0670 |

| **FIG S6:**  **Dark-Onset**  **Dark-Phase**  **Longitudinal** | **Repeated Measures 2-Way ANOVA**  **Mixed-Effects Analysis** | | |
| --- | --- | --- | --- |
|  | **ISO – SAL – CFA Weeks 1-3** | **ISO – SAL – CFA Week 1** | **CFA Weeks 1-3** |
| S6A  Awake Duration | Time F_(4.978,34.85)_=3.582, p=0.0103  Sex F_(1,7)_=6.634, p=0.0367  Time x Sex F_(29,203)_=1.396, p=0.0961 | Time F_(4.329,30.30)_=5.215, p=0.0021  Sex F_(1,7)_=7.383, p=0.0299  Time x Sex F_(17,119)_=0.8588, p=0.6228 | Time F_(5.052,35.36)_=6.133, p=0.0003  Sex F_(1,7)_=6.138, p=0.0424  Time x Sex F_(17,119)_=2.538, p=0.0018 |
| S6B  REM Duration | Time F_(3.907,27.35)_=6.887, p=0.0006  Sex F_(1,7)_=16.54, p=0.0048  Time x Sex F_(29,203)_=2.063, p=0.0020 | Time F_(3.637,25.46)_=8.815, p=0.0002  Sex F_(1,7)_=14.19, p=0.0070  Time x Sex F_(17,119)_=2.548, p=0.0017 | Time F_(3.094,21.66)_=12.26, p<0.0001  Sex F_(1,7)_=16.60, p=0.0047  Time x Sex F_(17,119)_=2.356, p=0.0038 |
| S6C  SWS Duration | Time F_(4.276,29.93)_=2.725, p=0.0449  Sex F_(1,7)_=7.156, p=0.0318  Time x Sex F_(29.203)_=1.325, p=0.1345 | Time F_(3.666,25.66)_=3.828, p=0.0163  Sex F_(1,7)_=8.592, p=0.0220  Time x Sex F_(17,119)_=0.7307, p=0.7661 | Time F_(4.777,33.44)_=4.762, p=0.0024  Sex F_(1,7)_=6.034, p=0.0437  Time x Sex F_(17,119)_=2.486, p=0.0022 |
| S6D  Awake Bouts | Time F_(4.505,31.53)_=3.878, p=0.0091  Sex F_(1,7)_=5.700, p=0.0483  Time x Sex F_(29,203)_=1.328, p=0.1325 | Time F_(4.011,28.07)_=5.600, p=0.0019  Sex F_(1,7)_=2.580, p=0.1523  Time x Sex F_(17,119)_=1.345, p=0.1771 | Time F_(4.572,32.00)_=5.163, p=0.0018  Sex F_(1,7)_=9.711, p=0.0169  Time x Sex F_(17,119)_=1.288, p=0.2119 |
| S6E  REM Bouts | Time F_(4.347,30.43)_=3.874, p=0.0101  Sex F_(1,7)_=1.564, p=0.2512  Time x Sex F_(29.203)_=1.536, p=0.0468 | Time F_(4.908,34.36)_=5.322, p=0.0011  Sex F_(1,7)_=0.8644, p=0.3834  Time x Sex F_(17,119)_=2.078, p=0.0119 | Time F_(4.373,30.61)_=5.381, p=0.0017  Sex F_(1,7)_=4.041, p=0.0843  Time x Sex F_(17,119)_=1.509, p=0.1026 |
| S6F  SWS Bouts | Time F_(3.737,36.16)_=1.116, p=0.3680  Sex F_(1,7)_=6.024, p=0.0438  Time x Sex F_(29.203)_=1.179, p=0.2526 | Time F_(3.445,24.11)_=1.290, p=0.3012  Sex F_(1,7)_=3.509, p=0.1032  Time x Sex F_(17,119)_=0.4354, p=0.9739 | Time F_(4.116,28.81)_=1.983, p=0.1221  Sex F_(1,7)_=5.234, p=0.0560  Time x Sex F_(17,119)_=2.0001, p=0.0162 |

| **FIG 6:**  **Temperature** | **RM 1-Way ANOVA** | **Multiple Comparisons** | **1-sample T-Test** | **2-Way ANOVA** | **Sidak’s Multiple Comparisons** |
| --- | --- | --- | --- | --- | --- |
| 6A  Light-Onset  Light Phase | F_(1.601,16.01)_=29.35, p<0.0001 | ISO vs. SAL p=0.0786  ISO vs. CFA p<0.0001  SAL vs. CFA p=0.0024 | ISO t_10_=1.717, p=0.1167  SAL t_10_=4.477, p=0.0012  CFA t_10_=7.023, p<0.0001 | Time F_(1.603,14.43)_=34.25, p<0.0001  Sex F_(1,9)_=0.4374, p=0.5249  Time x Sex F_(2,18)_=3.450, p=0.0539 | male vs. female:  ISO p=0.9624  SAL p>0.9999  CFA p=0.3501  male:  ISO vs. SAL p=0.6055  ISO vs. CFA p=0.0122  SAL vs. CFA p=0.1277  female:  ISO vs. SAL p=0.0942  ISO vs. CFA p=0.0013  SAL vs. CFA p=0.0165 |
| 6B  Light-Onset  Dark Phase | F_(1.932,19.32)_=35.72, p<0.0001 | ISO vs. SAL p=0.6461  ISO vs. CFA p<0.0001  SAL vs. CFA p=0.0002 | ISO t_10_=3.495, p=0.0058  SAL t_10_=6.000, p=0.0001  CFA t_10_=10.56, p<0.0001 | Time F_(1.955,17.59)_=33.11, p<0.0001  Sex F_(1,9)_=1.020, p=0.3390  Time x Sex F_(2,18)_=0.5006, p=0.6144 | male vs. female:  ISO p=0.8316  SAL p=0.9721  CFA p=0.8064  male:  ISO vs. SAL p=0.0463  ISO vs. CFA p=0.0132  SAL vs. CFA p=0.0532  female:  ISO vs. SAL p=0.9853  ISO vs. CFA p=0.0066  SAL vs. CFA p=0.0073 |
| 6C  Dark-Onset  Light Phase | F_(1.715,13.72)_=20.18, p=0.0001 | ISO vs. SAL p=0.1667  ISO vs. CFA p=0.0001  SAL vs. CFA p=0.0144 | ISO t_8_=3.473, p=0.0084  SAL t_8_=6.292, p=0.0002  CFA t_8_=9.640, p<0.0001 | Time F_(1.789,12.53)_=20.08, p=0.0002  Sex F_(1,7)_=0.02368, p=0.8820  Time x Sex F_(2,14)_=1.119, p=0.3542 | male vs. female:  ISO p=0.6056  SAL p=0.7033  CFA p=0.9901  male:  ISO vs. SAL p=0.8899  ISO vs. CFA p=0.0109  SAL vs. CFA p=0.1178  female:  ISO vs. SAL p=0.1672  ISO vs. CFA p=0.0145  SAL vs. CFA p=0.1755 |
| 6d  Dark-Onset  Dark Phase | F_(1.999,15.99)_=5.190, p=0.0183 | ISO vs. SAL p=0.0457  ISO vs. CFA p=0.9399  SLA vs. CFA p=0.0700 | ISO t_8_=2.719, p=0.0263  SAL t_8_=5.111, p=0.0009  CFA t_8_=2.959, p=0.0182 | Time F_(1.978,13.84)_=4.743, p=0.0273  Sex F_(1,7)_=0.03957, p=0.8480  Time x Sex F_(2,14)_=0.6195, p=0.5523 | male vs. female:  ISO p=0.9171  SAL p=0.9756  CFA p=0.9361  male:  ISO vs. SAL p=0.2882  ISO vs. CFA p=0.6400  SAL vs. CFA p=0.5222  female:  ISO vs. SAL p=0.2307  ISO vs. CFA p=0.9099  SAL vs. CFA p=0.1576 |
